# Forkhead box O6 (FoxO6) promotes cardiac pathological remodeling and dysfunction by activating Kif15–TGF‐β1 under aggravated afterload

**DOI:** 10.1002/mco2.383

**Published:** 2023-10-03

**Authors:** Bing Zhang, Lei Shi, Yanzhen Tan, Yenong Zhou, Jun Cui, Yujie Song, Yingying Liu, Miao Zhang, Weixun Duan, Zhenxiao Jin, Jincheng Liu, Dinghua Yi, Yang Sun, Wei Yi

**Affiliations:** ^1^ Department of Cardiovascular Surgery Xijing Hospital The Fourth Military Medical University Xi'an China; ^2^ Department of Geriatrics Xijing Hospital The Fourth Military Medical University Xi'an China

**Keywords:** cardiac fibrosis, FoxO6, heart failure, Kif15, TGF‐β1

## Abstract

Pathological cardiac hypertrophy exhibits complex and abnormal gene expression patterns and progresses to heart failure. Forkhead box protein O6 (FoxO6) is a key transcription factor involved in many biological processes. This study aimed to explore the role of FoxO6 in cardiac hypertrophy. Three groups of mice were established: wild‐type, FoxO6 knockout, and FoxO6‐overexpressing. The mice received daily administration of angiotensin‐II (Ang‐II) or saline for 4 weeks, after which they were examined for cardiac hypertrophy, fibrosis, and function. Elevated cardiac expression of FoxO6 was observed in Ang‐II‐treated mice. FoxO6 deficiency attenuated contractile dysfunction and cardiac remodeling, including cardiomyocyte hypertrophy and fibroblast proliferation and differentiation. Conversely, FoxO6 overexpression aggravated the cardiomyopathy and heart dysfunction. Further studies identified kinesin family member 15 (Kif15) as downstream molecule of FoxO6. Kif15 inhibition attenuated the aggravating effect of FoxO6 overexpression. In vitro, FoxO6 overexpression increased Kif15 expression in cardiomyocytes and elevated the concentration of transforming growth factor‐β1 (TGF‐β1) in the medium where fibroblasts were grown, exhibiting increased proliferation and differentiation, while FoxO6 knockdown attenuated this effect. Cardiac‐derived FoxO6 promoted pathological cardiac remodeling induced by aggravated afterload largely by activating the Kif15/TGF‐β1 axis. This result further complements the mechanisms of communication among different cells in the heart, providing novel therapeutic targets for heart failure.

## Graphical abstract

Pressure overload is a key promoter of hypertensive cardiac remodeling characterized by left ventricular hypertrophy and fibrosis. Cardiac fibrosis is characterized by abnormal gene expression patterns that can progress to heart failure and is identified as a vital therapeutic target for heart failure. The transcription factor FoxO6 is involved in many biological processes. The regulatory role of FoxO6 in cardiac diseases remains unclear.

This study elucidated for the first time that. FoxO6 is an essential transcription factor that shows a strong association with the progression of cardiac disease. FoxO6 promotes pathological cardiac fibrosis and left ventricular contractile dysfunction by activating Kif15 and TGF‐β1 secretion in cardiomyocytes under pressure overload, which facilitates the proliferation and differentiation of fibroblasts. FoxO6 expression knockout leads to anti‐fibrosis effect and conserves cardiac function.

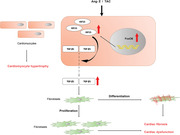

## 1 INTRODUCTION

Chronic heart failure (HF) can be caused by a variety of heart diseases and has a high mortality, which is a huge burden on global health.^1^ Although cardiomyocytes (CMs) form most of the cell mass of the heart, non‐CMs outnumber CMs.^2^ The interactions and cooperation, both normal and pathophysiological, among these cell types are poorly understood. Cardiac remodeling, a change in myocardial structure to compensate for cardiac insufficiency, is significantly associated with developing HF and consists of various pathophysiological manifestations, including cardiac hypertrophy, cardiac fibrosis, and angiogenesis.^3^ Cardiac fibrosis is also significantly linked to overall mortality and hospitalization because of its impact on exaggerating mechanical myocardial stiffness and disorganizing the cardiac systolic function.^4^ Although inhibiting cardiac fibrosis remains a key therapeutic direction for patients with HF, no effective molecular target has been identified.

Various transcriptional regulatory factors and signaling pathways are involved in the pathogenesis of cardiac remodeling.^5^ The Forkhead box O (FoxO) protein family (including FoxO1, FoxO3, FoxO4, and FoxO6) has a common “fork” or “winged” spiral structure domain that allows for their binding to nuclear DNA. FoxOs are closely associated with regulating cellular proliferation, apoptosis, autophagy, metabolism, differentiation, and inflammation.^6^ FoxO expression and activation have protective functions in models of diabetic cardiomyopathy, ischemic heart disease, and cardiac hypertrophy.^7^, ^8^, ^9^ Regulating these proteins is a promising method for the treatment of cardiac diseases. However, there is still no definitive treatment for HF and myocardial remodeling with FoxOs.

FoxO6 is the newest member, and relatively little research has been done on it. Regarding cardiovascular disease, FoxO6 damages hypoxic CMs via promoting apoptosis and oxidative stress.^10^ In other areas, FoxO6 is clearly involved in vaious physiological processes, including cell proliferation, energy metabolism, redox reactions, etc. FoxO6 plays a vital role in oxidative stress during cell proliferation.^11^ FoxO6 also modulates oxidative metabolism in muscle cells.^12^ Combined with the excessive proliferation of fibroblasts and abnormal energy metabolism in CMs during HF, we suspect that FoxO6 plays an important role in heart disease.

In this study, we created a mouse model of pathological cardiac hypertrophy by angiotensin‐II (Ang‐II) treatment and transverse aortic constriction (TAC) to explore the function of FoxO6 in the modulation of cardiac function and remodeling. We also characterized the signaling pathways regulated by FoxO6 in cardiac hypertrophy. Our findings are intended to provide novel research ideas and therapeutic targets for HF.

## 2 RESULTS

### 2.1 Upregulation of FoxO6 expression in hypertrophic hearts and Ang‐II‐treated neonatal rat CMs

**FIGURE 1:**
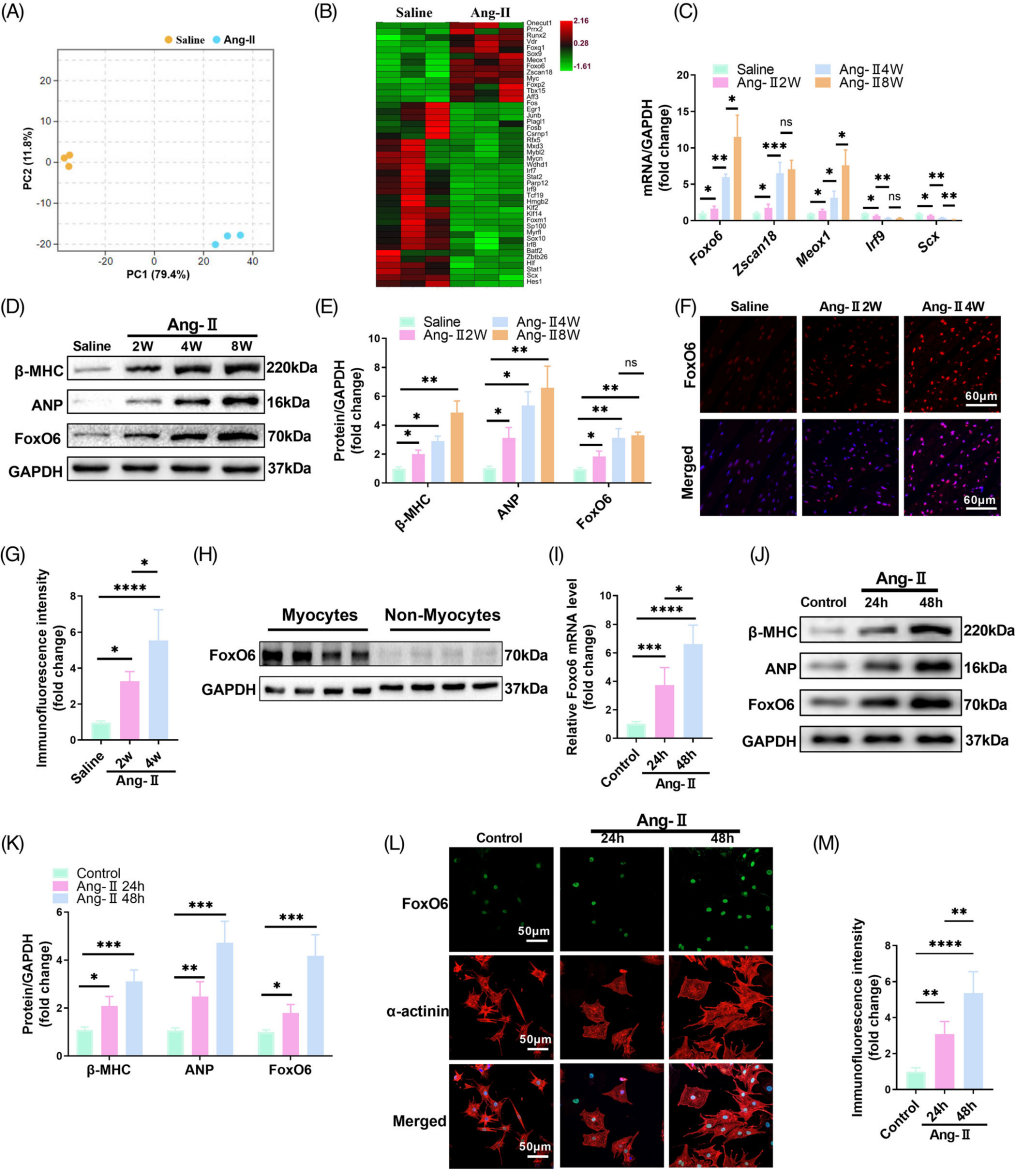
Forkhead box protein O6 (FoxO6) expression was upregulated in myocardium and cardiomyocytes (CMs) exposed to angiotensin‐II (Ang‐II). (A) Principal component analysis (PCA) of sham and Ang‐II groups. (B) Heatmap of differentially expressed transcription factors between sham and Ang‐II groups. (C) Cardiac mRNA expression levels of genes encoding FoxO6, Zscan18, Meox1, Irf9, and Scx (*n* = 6 mice per group). (D) Typical western blots indicating myosin heavy chain β (β‐MHC), atrial natriuretic peptide (ANP), and FoxO6 expression levels in myocardium of mice after Ang‐II treatment (*n* = 6). (E) Cardiac protein expression levels of β‐MHC, ANP, and FoxO6 in mice after Ang‐II treatment (*n* = 6). (F) Immunostaining of murine myocardium sections to exhibit the expression of FoxO6 (red), DAPI (blue) was used to stain nuclei. (G) FoxO6 immunofluorescence intensity (*n* = 6). (H) Cardiac protein expression levels of FoxO6 in CMs and non‐CMs from murine myocardium (*n* = 4). (I) Cardiomyocyte mRNA expression levels of gene encoding FoxO6 (*n* = 5). (J) Typical western blots indicating β‐MHC, ANP, and FoxO6 levels in neonatal rat CMs (NRCMs) from indicated groups. (K) Cardiomyocyte protein expression levels of β‐MHC, ANP, and FoxO6 (*n* = 5). (L) Immunostaining of NRCMs after Ang‐II treatment to exhibit the expression of FoxO6 (green), α‐actinin (red), and DAPI (blue). (M) FoxO6 immunofluorescence intensity (*n* = 5). Data were analyzed by one‐way analysis of variance (ANOVA). ^*^ *p* < 0.05, ^**^ *p* < 0.01, ^***^ *p* < 0.001, ^****^ *p* < 0.0001. Statistics are carried out as mean ± standard deviation (SD).

We performed a transcriptome analysis on heart tissues from the Ang‐II‐ and saline‐treated mice and observed that gene expression differed significantly between the two groups (Figure 1A). We found many transcriptional regulatory factors with significantly altered expression levels induced by Ang‐II administration (Figure 1B). Ang‐II significantly increased *Foxo6*, *Zscan18*, and *Meox1* mRNA levels and decreased *Irf9* and *Scx* mRNA levels 2, 4, and 8 weeks after treatment, dependent on time (Figure 1C). Among these five potentially critical factors, only FoxO6 protein levels also increased significantly with time after Ang‐II treatment, paralleled by raised levels of the indices of cardiac hypertrophy, specifically, myosin heavy chain β (β‐MHC) and atrial natriuretic peptide (ANP) (Figure 1D,E). The immunofluorescence intensity of FoxO6 increased with time after Ang‐II treatment in TAC‐operated versus sham‐operated wild‐type (WT) mice (Figure 1F,G). Western blotting indicated significantly greater expression of FoxO6 in CMs than in non‐CMs (Figure 1H). In the Ang‐II‐treated neonatal rat CMs (NRCMs) after 24 and 48 h, FoxO6 expression (both mRNA and protein) was significantly elevated, in parallel with those of β‐MHC and ANP (Figure 1I–K). FoxO6 immunofluorescence intensity was also enhanced after Ang‐II treatment in NRCMs (Figure 1L,M).

### 2.2 FoxO6 deficiency alleviated heart dysfunction and cardiac pathological remodeling after Ang‐II treatment

**FIGURE 2:**
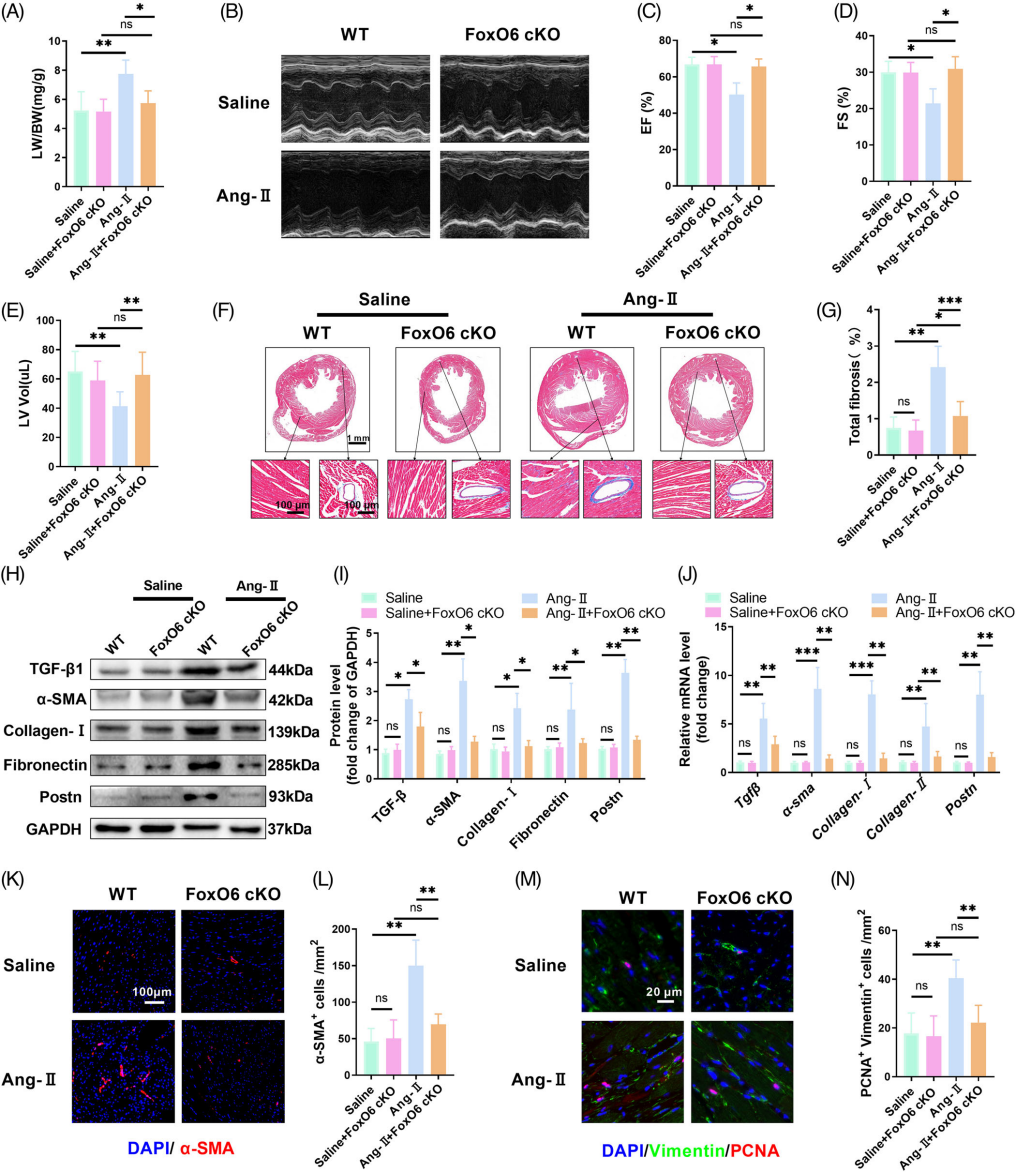
Cardiac‐specific knockout of Forkhead box protein O6 (FoxO6) inhibited angiotensin‐II (Ang‐II)‐induced cardiac fibrosis. (A) Lung‐to‐body weight (LW/BW) ratio of mice (*n* = 5 mice per group). (B) Echocardiography of mice. (C–E) Left ventricular ejection fraction (LVEF), fraction shortening (FS)%, and left ventricle volume (LV vol) determined using echocardiography (*n* = 8). (F) Typical images of murine heart sections stained with Masson's trichrome stain, perivascular area, and interstitial area were amplified. (G) Degree of left ventricular (LV) fibrosis evidenced by collagen volume (*n* = 7). (H) Typical western blots indicating transforming growth factor‐β1 (TGF‐β1), α‐SMA, collagen I, fibronectin, and Postn in murine myocardium. (I) Cardiac protein expression levels of TGF‐β1, α‐SMA, collagen I, fibronectin, and Postn in murine myocardium (*n* = 6). (J) Cardiac mRNA expression levels of genes encoding fibrotic markers TGF‐β1, α‐SMA, collagen I, collagen III, and Postn in each group (*n* = 6). (K) Immunostaining of murine myocardium sections to exhibit the expression of α‐SMA (red). (L) Number of α‐SMA‐positive cells (*n* = 6). (M) Immunostaining of murine myocardium sections to exhibit the expression of vimentin (green) and proliferating cell nuclear antigen (PCNA) (red). (N) Number of vimentin‐ and PCNA‐positive cells (*n* = 6). Data were analyzed by one‐way analysis of variance (ANOVA). ^*^ *p* < 0.05, ^**^ *p* < 0.01, ^***^ *p* < 0.001, ^****^ *p* < 0.0001. Statistics are carried out as mean ± standard deviation (SD).

We generated FoxO6 CM‐specific‐knockout (FoxO6 cKO) mice. The 8–10‐week‐old FoxO6 cKO mice showed no apparent abnormalities compared with WT mice. FoxO6 expression was obviously knocked down in the hearts of FoxO6 cKO mice (Figure S1A), and there was no significant expression in CMs (Figure S1B). In addition to the myocardial tissue, the expression of FoxO6 in other tissues, including the brain, liver, and kidney, did not vary from that in WT mice (Figure S1C). Four weeks after saline or Ang‐II was administered, echocardiography and histological evaluations confirmed the successful establishment of the cardiac hypertrophy model. The heart‐to‐body weight (HW/BW) ratio was significantly decreased in the FoxO6 cKO mice compared to WT mice after Ang‐II treatment (Figure S2A). The lung‐to‐body weight (LW/BW) ratio was lower in FoxO6 cKO mice than in WT mice after Ang‐II treatment (Figure 2A), indicating that pulmonary edema caused by cardiac dysfunction was mitigated. After Ang‐II treatment, left ventricular (LV) wall thickness and mass increased significantly, as evidenced by increased interventricular septal thickness at end diastole, interventricular septal thickness at end systole, left ventricular posterior wall at end diastole (LVPWd), and left ventricular posterior wall at end systole (LVPWs). FoxO6 deficiency remarkably reduced the degree of cardiac hypertrophy (Figures 2B and S2B–F).

In our study, Ang‐II treatment caused obvious cardiac systolic dysfunction, and FoxO6 cardiac‐specific deficiency significantly improved cardiac function (Figure 2C,D). LV volume decreased significantly in Ang‐II‐treated WT and FoxO6 cKO mice owing to LV centripetal hypertrophy (Figure 2E). The gross heart was inspected under the microscope, and sections were stained with hematoxylin–eosin (HE) and wheat germ agglutinin stained to assess the level of cardiac hypertrophy. Hypertrophy was significantly worse in Ang‐II‐treated WT mice than in the controls, which was attenuated by FoxO6 knockout, as shown by the average cross‐sectional area (Figure S2G,H). Significant upregulation of hypertrophy markers, including *β‐mhc*, *Anp*, regulator of calcineurin 1.4 (*Rcan1.4*), α‐sarcomeric actin (*Acta‐1*), and brain natriuretic peptide (*Bnp*), was seen after 4 weeks of Ang‐II treatment compared with the sham‐operated group, while FoxO6 silencing reversed the increases in *Anp* and *Bnp* (Figure S2I). Notably, cardiac fibrosis induced by Ang‐II treatment was significantly alleviated by FoxO6 deficiency, as evidenced by LV collagen volume (Figure 2F,G). The expression of genes associated with fibrosis, including *Tgf‐β1*, *α‐sma*, *Collagen‐I* (*Col‐I*), *Fibronectin*, and *Postn*, was elevated in the hearts of Ang‐II‐treated mice after Ang‐II treatment, while FoxO6 deficiency significantly weakened this effect (Figure 2H,I). Similar results were observed using quantitative polymerase chain reaction (qPCR) (Figure 2J).

Fibroblast (FB) differentiation and proliferation were measured by immunofluorescence staining. The number of α‐smooth muscle actin (SMA)‐positive cells increased after Ang‐II treatment and decreased in Ang‐II + FoxO6 cKO mouse hearts (Figure 2K,L). The number of proliferating cell nuclear antigen (PCNA)‐positive and vimentin co‐positive cells, reflecting fibroblast proliferation, also increased significantly after Ang‐II treatment, and FoxO6 deficiency reversed this effect (Figure 2M,N).

### 2.3 FoxO6 overexpression exacerbated left ventricular systolic dysfunction and cardiac fibrotic remodeling after Ang‐II treatment

**FIGURE 3:**
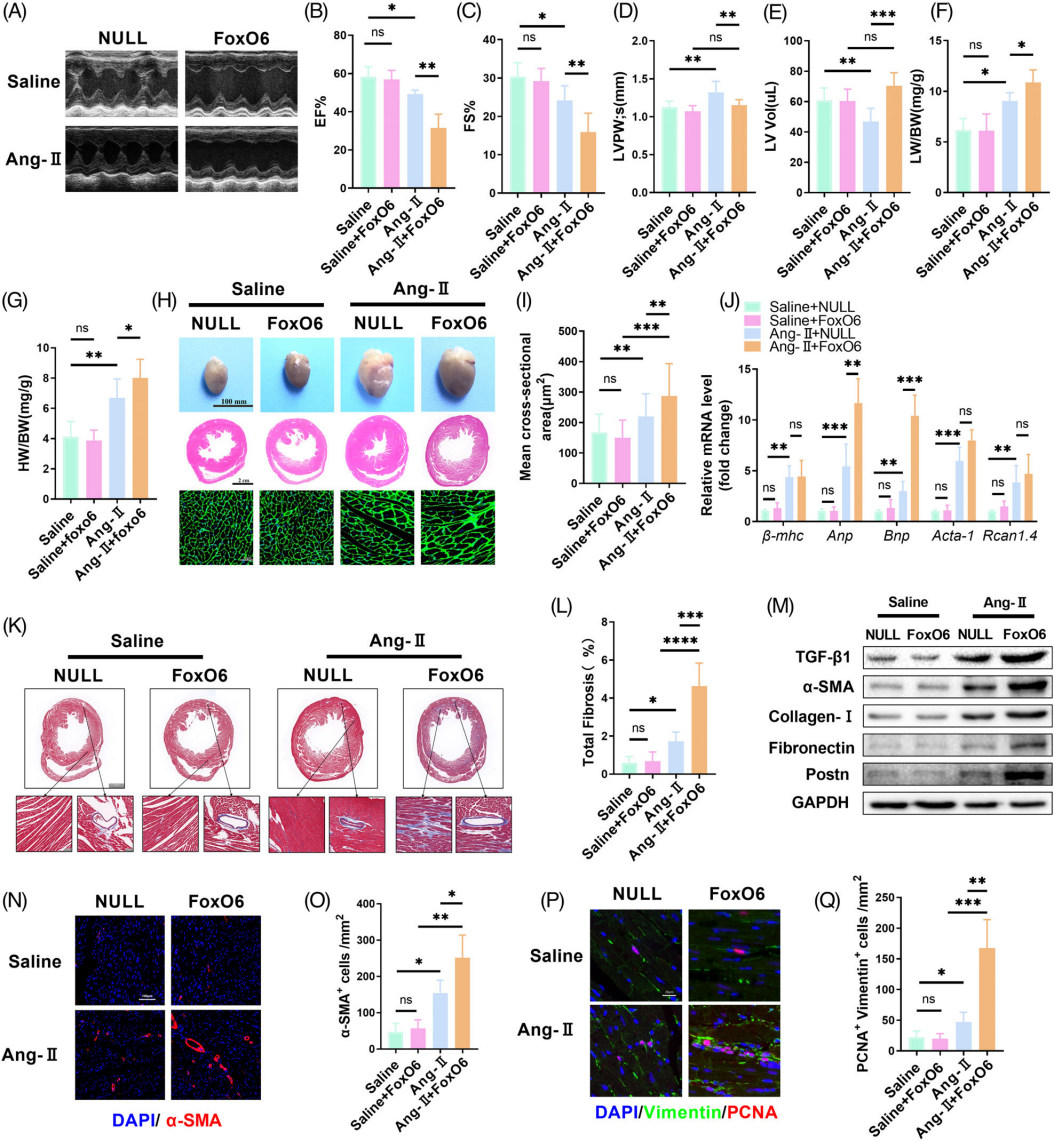
Myocardial overexpression of Forkhead box protein O6 (FoxO6) aggravated angiotensin‐II (Ang‐II)‐induced myocardial fibrosis and cardiac dysfunction. (A) Echocardiography of mice. (B–E) Ejection fraction (EF)%, fraction shortening (FS)%, left ventricular posterior walls (LVPWs), and left ventricle volume (LV vol), respectively, determined using echocardiography (*n* = 8). (F) Lung‐to‐body weight (LW/BW) ratio of mice (*n* = 6). (G) HW/BW ratio of mice (*n* = 5). (H) Gross anterior view of murine hearts and typical images of sections stained with hematoxylin and eosin (HE) and wheat germ agglutinin (WGA). (I) Analysis of cardiomyocytes (CMs) cross‐sectional area (*n* ≥ 100 cells/group). (J) Cardiac mRNA expression levels of genes encoding hypertrophic markers myosin heavy chain β (β‐MHC), atrial natriuretic peptide (ANP), brain natriuretic peptide (BNP), Acta‐1, and Rcan1.4 in each group (*n* = 6). (K) Typical images of murine heart sections stained with Masson's trichrome stain, perivascular area, and interstitial area were amplified. (L) Degree of left ventricular (LV) fibrosis evidenced by collagen volume (*n* = 7). (M) Cardiac protein expression levels of transforming growth factor‐β1 (TGF‐β1), α‐SMA, collagen I, fibronectin, and Postn levels in murine myocardium (*n* = 6). (N) Immunostaining of murine myocardium sections to exhibit the expression of α‐SMA (red). (O) Number of α‐SMA‐positive cells (*n* = 6). (P) Immunostaining of murine myocardium sections to exhibit the expression of vimentin (green) and proliferating cell nuclear antigen (PCNA) (red). (Q) Number of vimentin‐ and PCNA‐positive cells (*n* = 6). Data were analyzed by one‐way analysis of variance (ANOVA). ^*^ *p* < 0.05, ^**^ *p* < 0.01, ^***^ *p* < 0.001, ^****^ *p* < 0.0001. Statistics are carried out as mean ± standard deviation (SD).

We generated a mouse model of CM‐specific FoxO6‐overexpressing myocardial tissue to clarify further the effect of FoxO6 on the progression of pathological myocardial remodeling (Figure S3A,B). We also constructed FoxO6‐overexpressing cell model and confirmed the effects in FBs (Figure S3C,D) and CMs (Figure S3E,F). Ang‐II treatment caused specific cardiac dysfunction, but FoxO6 overexpression further caused severe cardiac dysfunction, as evidenced by decreased LV ejection fraction (EF) and fraction shortening (FS) (Figure 3A–C). Although FoxO6 overexpression did not alter the degree of Ang‐II‐induced hypertrophy (Figure S4A–D), it reduced LVPW (Figure 3D). The LV volume was significantly greater in FoxO6‐overexpressing mouse hearts than in WT mouse hearts after Ang‐II treatment (Figure 3E), together with higher LW/BW ratios (Figure 3F), indicating that pulmonary edema caused by cardiac dysfunction was aggravated. There was a significant rise in the HW/BW ratio in FoxO6‐overexpressing animals compared to WT animals following Ang‐II treatment (Figure 3G). The degree of cardiac hypertrophy was also obviously aggravated in Ang‐II‐treated FoxO6‐overexpressing mice (Figure 3H–I). FoxO6 overexpression promoted the upregulation of *Anp* and *Bnp* mRNA levels following Ang‐II treatment compared with those in WT mice, further suggesting an exacerbated effect on HF (Figure 3J).

Ang‐II‐induced cardiac fibrosis was aggravated by FoxO6 overexpression, as evidenced by LV collagen volume (Figure 3K,L) and expression of fibrotic genes detected by western blotting and qPCR (Figures 3M and S4E). Fibroblast differentiation and proliferation were further increased, as evidenced by α‐SMA‐positive and PCNA‐positive and vimentin co‐positive cell counts in mouse heart tissues from the FoxO6 + Ang‐II group compared with those from the NULL + Ang‐II group (Figure 3N–Q). Although FoxO6 overexpression can significantly promote CM hypertrophy and increase heart size, there is no significant increase in LV wall thickness. In addition, significant expansion of LV volume, significant reduction of cardiac systolic function, and severe myocardial fibrosis suggest that FoxO6 overexpression plays a critical role in promoting HF.

### 2.4 FoxO6 overexpression promoted expression of genes associated with extracellular matrix synthesis and development

**FIGURE 4:**
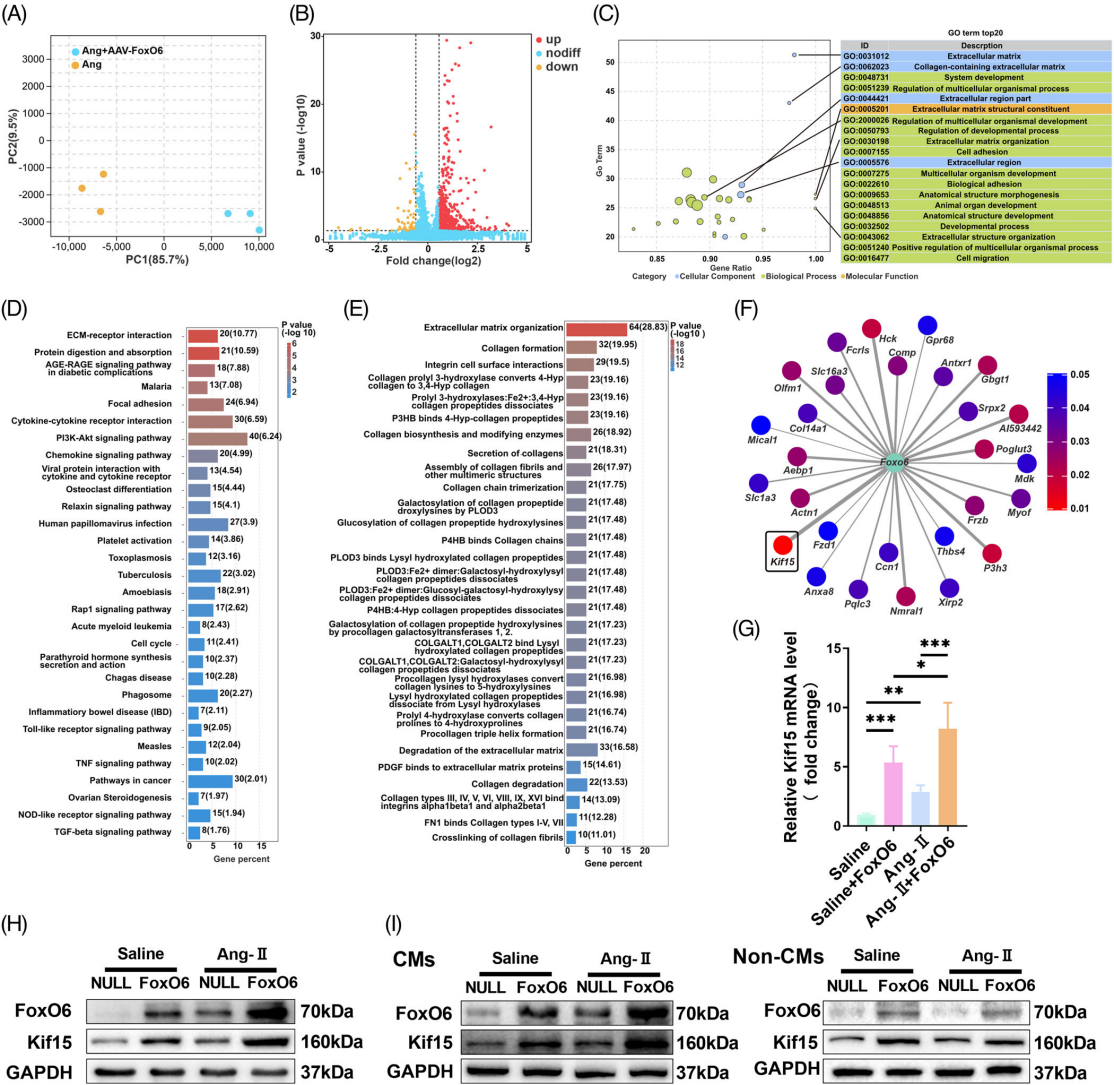
Overexpression of Forkhead box protein O6 (FoxO6) activated extracellular matrix remodeling and upregulated Kif15 expression. (A) Principal component analysis (PCA) of angiotensin‐II (Ang‐II) and Ang‐II + adeno‐associated virus (AAV)‐FoxO6 groups. (B) Volcano map showing great differences in gene expression pattern between Ang‐II and Ang‐II + AAV‐FoxO6 groups. (C) Gene Ontology (GO) enrichment analysis of top 20 differentially expressed genes (DEGs). (D) Pathways significantly enriched in myocardial tissue after FoxO6 overexpression. (E) Reactome analysis of significantly enriched reactions in myocardial tissue after FoxO6 overexpression. (F) Kif15 is gene with most significant increases in expression level. (G) Cardiac mRNA expression levels of gene encoding Kif15 (*n* = 6). (H) Cardiac protein expression levels of FoxO6 and Kif15 levels in murine myocardium (*n* = 6). (I) Cardiac protein expression levels of FoxO6 and Kif15 in cardiomyocytes (CMs) and non‐CMs from murine myocardium (*n* = 6). Data were analyzed by one‐way analysis of variance (ANOVA). ^*^ *p* < 0.05, ^**^ *p* < 0.01, ^***^ *p* < 0.001, ^****^ *p* < 0.0001. Statistics are carried out as mean ± standard deviation (SD).

To further characterize the mechanism by which FoxO6 regulates cardiac function and remodeling, we sequenced RNA from the heart tissues of the FoxO6 + Ang‐II and NULL + Ang‐II groups. Significant differences were observed in gene expression patterns of the myocardial tissues between these two groups (Figure 4A,B). According to Gene Ontology (GO) terms, we identified two groups of differentially expressed genes (DEGs). The top 20 DEGs were related to extracellular matrix (ECM) remodeling and formation (Figure 4C). The ECM–receptor interaction was the most significant signaling pathway in the myocardium of mice after FoxO6 overexpression (Figure 4D). Reactome analysis also confirmed that FoxO6 overexpression expedited ECM organization and collagen formation (Figure 4E). To determine the downstream regulatory mechanisms, we identified the first molecules with the most obvious changes. FoxO6 overexpression upregulated the expression of *Kif15* (Figure 4F), which was confirmed by qPCR and western blotting (Figure 4G,H). In FoxO6 cKO mice, Kif15 expression decreased significantly in heart tissues, regardless of the treatment (Figure S4F–H).

FoxO6 expression was significantly elevated in Ang‐II‐treated CMs relative to that in non‐CMs, and the trend of Kif15 expression was in line with that of FoxO6. No change in FoxO6 and Kif15 expression was found in non‐CMs following Ang‐II treatment, regardless of infection with the FoxO6 adenovirus (Ad) (Figure 4I).

### 2.5 Kif15 is a downstream factor of FoxO6 that promotes Ang‐II‐induced cardiac fibrosis and dysfunction

**FIGURE 5:**
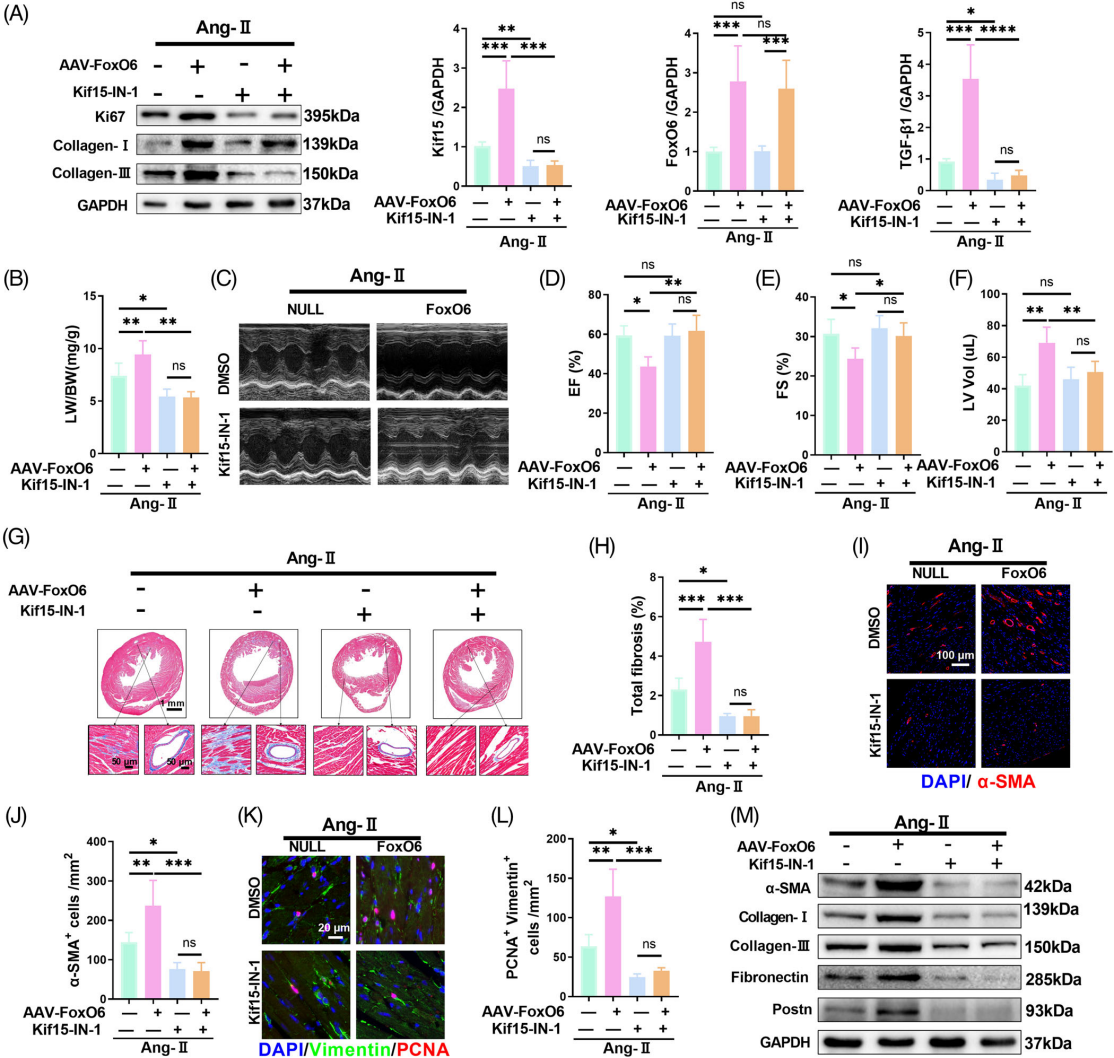
Inhibition of Kif15 eliminated pro‐fibrotic and adverse cardiac function effects caused by Forkhead box protein O6 (FoxO6) overexpression. (A) Cardiac protein expression levels of FoxO6, Kif15, and transforming growth factor‐β1 (TGF‐β1) in murine myocardium (*n* = 6). (B) Lung‐to‐body weight (LW/BW) ratio of mice (*n* = 6). (C) Echocardiography of mice. (D–F) Ejection fraction (EF)%, fraction shortening (FS)%, and left ventricle volume (LV vol), respectively, determined by echocardiography (*n* = 8). (G) Typical images of murine heart sections stained with Masson's trichrome stain, perivascular area and interstitial area were amplified. (H) Degree of LV fibrosis evidenced by collagen volume (*n* = 7). (I) Immunostaining of murine myocardium sections to exhibit the expression of α‐SMA (red). (J) Number of α‐SMA‐positive cells (*n* = 6). (K) Immunostaining of murine myocardium sections to exhibit the expression of vimentin (green) and proliferating cell nuclear antigen (PCNA) (red). (L) Number of vimentin‐ and PCNA‐positive cells (*n* = 6). (M) Cardiac protein expression levels of α‐SMA, collagen I, collagen III, fibronectin, and Postn in murine myocardium (*n* = 6). Data were analyzed by one‐way analysis of variance (ANOVA). ^*^ *p* < 0.05, ^**^ *p* < 0.01, ^***^ *p* < 0.001, ^****^ *p* < 0.0001. Statistics are carried out as mean ± standard deviation (SD).

To test whether Kif15 mediated FoxO6 actions on the Ang‐II‐treated myocardium, Kif15‐IN‐1 was used to suppress the expression of Kif15. Kif15‐IN‐1 inhibited kif15 expression, while FoxO6 expression was unchanged (Figure 5A). Furthermore, transforming growth factor‐β1 (TGF‐β1) expression was downregulated along with that of Kif15 (Figure 5A). In Ang‐II‐treated mice, the elevated LW/BW ratio in the FoxO6‐overexpressing group was significantly reduced by Kif15‐IN‐1 (Figure 5B). FoxO6 overexpression remarkably attenuated cardiac function in Ang‐II‐treated mice, while Kif15‐IN‐1 treatment blunted this effect (Figure 5C–E). Similarly, the effect of increased myocardial remodeling induced by FoxO6 overexpression was significantly attenuated by Kif15‐IN‐1, as indicated by LV volume (Figure 5F) and total fibrosis (Figure 5G,H). Fibroblast differentiation and proliferation induced by Ang‐II treatment were aggravated by FoxO6 overexpression. However, this effect was blunted by Kif15‐IN‐1 (Figure 5I–M). Finally, we detected fibrotic genes in different groups using western blotting and observed that the elevated expression of fibrotic genes in the FoxO6 + Ang‐II group was significantly reduced by IN‐1 treatment.

### 2.6 FoxO6 aggravated proliferation and differentiation of cardiac fibroblasts by increasing the secretion of TGF‐β1 in CMs

To determine the specific mechanism of FoxO6 in cardiac remodeling, we regulated FoxO6 expression in CMs and fibroblasts. We designed the FoxO6 siRNA sequences (Figure S5A) and confirmed their knockdown effect in CMs (Figure S5B,C) and FBs (Figure S5D,E). Ang‐II administration significantly increased the sizes of NRCMs, while FoxO6 knockdown attenuated this effect (Figure S6A,B). Furthermore, FoxO6 siRNA reduced the levels of β‐MHC, ANP, and TGF‐β1 in CMs treated with Ang‐II (Figure S6C,D). With Ad FoxO6 transfection and Ang‐II treatment, FoxO6 overexpression further exacerbated cardiac hypertrophy and increased the expression of β‐MHC, ANP, and TGF‐β1 (Figure S6E–H). The expression of Kif15 was observed to increase after Ang‐II administration and changed along with the expression of FoxO6 (Figure S6I,J). We regulated the expression of FoxO6 in TGF‐β‐treated fibroblasts and found no obvious change in α‐SMA expression (Figure S7).

**FIGURE 6:**
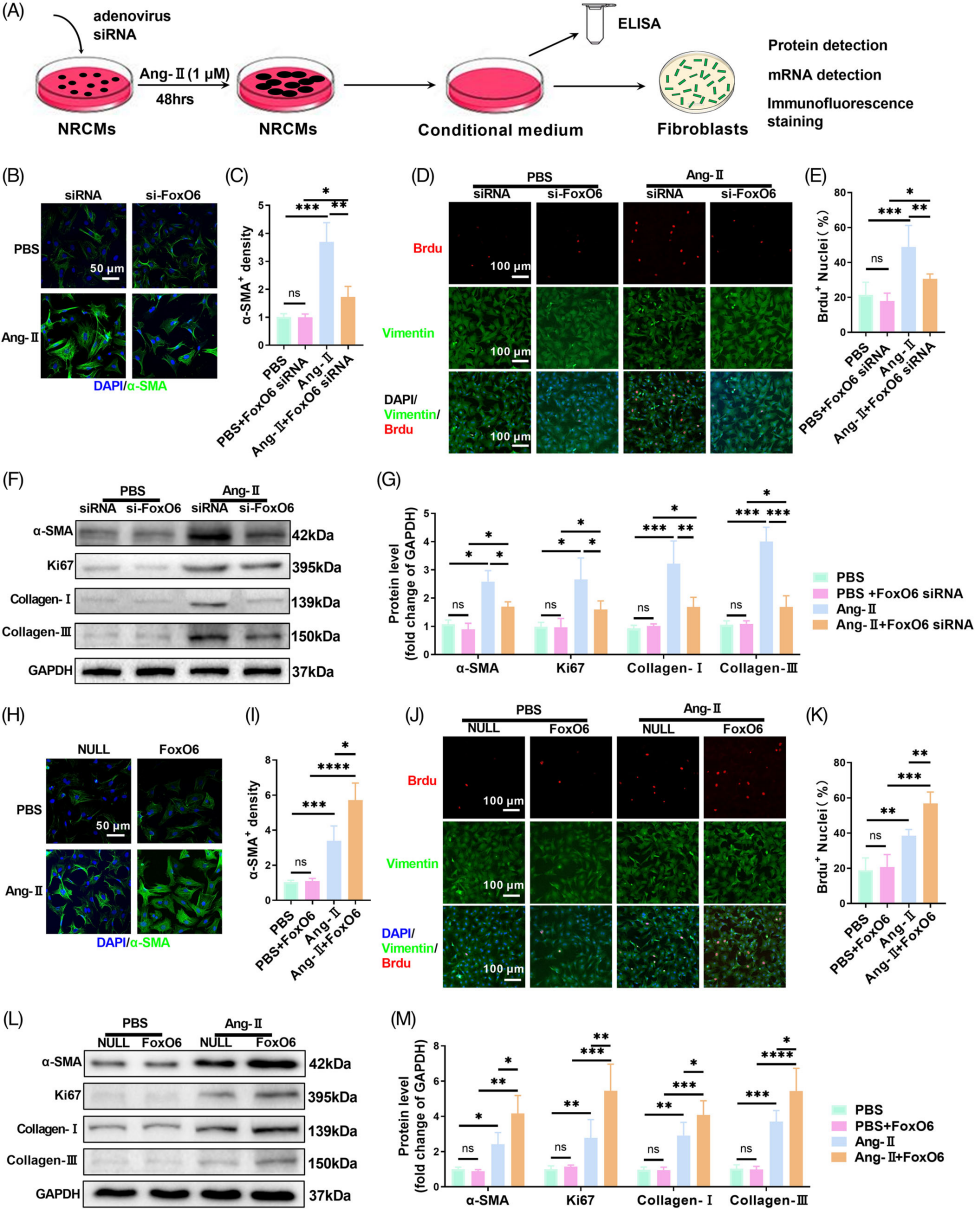
Give‐and‐loss function of Forkhead box protein O6 (FoxO6) in neonatal rat cardiomyocytes (NRCMs) regulated expression of transforming growth factor‐β1 (TGF‐β1) in culture medium and proliferation and differentiation of fibroblasts. (A) In vitro model: under angiotensin‐II (Ang‐II) stimulation, expression of FoxO6 in cardiomyocytes (CMs) was regulated, concentration of TGF‐β1 in culture medium of CMs in each group was detected, and same culture medium of each group was added to corresponding batch of fibroblast culture dishes to observe changes in each group. (B) Immunostaining of fibroblasts to exhibit the expression of α‐SMA (green). (C) α‐SMA immunofluorescence intensity (*n* = 5 samples per group). (D) Immunostaining of fibroblasts to exhibit the expression of vimentin (green), BrdU (red), and DAPI (blue). (E) Number of BrdU‐positive nuclei (*n* = 5). (F) Typical western blots indicating α‐SMA, Ki67, collagen I, and collagen III in fibroblasts. (G) FB protein expression levels of α‐SMA, Ki67, collagen I, and collagen III (*n* = 6). (H) Immunostaining of fibroblasts to exhibit the expression of α‐SMA (green). (I) α‐SMA immunofluorescence intensity (*n* = 5). (J) Immunostaining of fibroblasts to exhibit the expression of vimentin (green), BrdU (red), and DAPI (blue). (K) Number of BrdU‐positive nuclei (*n* = 5). (L) Typical western blots indicating α‐SMA, Ki67, collagen I, and collagen III in fibroblasts. (M) FB protein expression levels of α‐SMA, Ki67, collagen I, and collagen III (*n* = 6). Data were analyzed by one‐way analysis of variance (ANOVA). ^*^ *p* < 0.05, ^**^ *p* < 0.01, ^***^ *p* < 0.001, ^****^ *p* < 0.0001. Statistics are carried out as mean ± standard deviation (SD).

We inhibited the activity of Kif15 in NRCMs to verify the mechanism by which FoxO6 regulates CM hypertrophy. Kif15 inhibition indeed blocked the enhanced effect of FoxO6 overexpression on CM hypertrophy (Figure S8A,B) and inhibited protein expression of ANP and β‐MHC (Figure S8C,D). Since the expression of FoxO6 in myocardial fibroblasts does not affect its differentiation, we hypothesized that FoxO6 promotes fibroblast proliferation and differentiation by enhancing the communication between CMs and fibroblasts. To investigate this, we evaluated the effects of culturing fibroblasts with the culture medium of NRCMs incubated with Ang‐II for 48 h (Figure 6A). This showed that Ang‐II‐enhanced TGF‐β1 secretion by NRCMs, whereas FoxO6 siRNA attenuated this effect (Figure S9A), but FoxO6 overexpression enhanced it (Figure S9B). The NRCM culture medium significantly increased the degree of fibroblast differentiation, whereas this effect was reduced by FoxO6 silencing, as evidenced by α‐SMA immunofluorescence intensity (Figure 6B,C). Similarly, the degree of fibroblast proliferation was elevated by the NRCM culture medium but was inhibited by FoxO6 knockdown, as evidenced by the number of BrdU‐positive cells (Figure 6D,E).

We also detected fibrosis‐related genes, such as α‐SMA, collagen I, collagen III, and the proliferation‐related gene Ki67 by western blotting. The expression of these genes increased in fibroblasts cultivated in the NRCMs culture medium, but this effect was abolished in FoxO6‐silenced NRCMs incubated with Ang‐II (Figure 6F,G). In fibroblasts cultured in the spent medium of NRCMs overexpressing FoxO6 and treated with Ang‐II, the differentiation, and proliferation of fibroblasts were further intensified (Figure 6H–K), and the levels of α‐SMA, Ki67, Col‐I, and Col‐III were further enhanced (Figure 6L,M).

### 2.7 Kif15 and FoxO6 act synergistically in promoting TGF‐β1 secretion in CMs

We tested the role of Kif15 in determining TGF‐β1 in CMs under FoxO6 overexpression. We observed similar immunofluorescence intensities for FoxO6 and Kif15 in CMs (Figure S10). We administered Kif15‐IN‐1 to FoxO6‐overexpressing or control Ang‐II‐treated NRCMs and used the culture medium to cultivate fibroblasts. We found that the enhanced differentiation and proliferation of fibroblasts caused by FoxO6 overexpression were abolished by Kif15‐IN‐1, as evidenced by α‐SMA immunofluorescence intensity and the numbers of BrdU‐positive cells (Figure S11A–D). The elevated expression of α‐SMA, Ki67, Col‐I, and Col‐III induced by FoxO6 overexpression was also blunted by Kif15‐IN‐1 (Figure S11E,F). Lastly, we found that Kif15‐IN‐1 abolished the effect of FoxO6 overexpression in promoting TGF‐β1 secretion (Figure S11G).

### 2.8 FoxO6 deficiency attenuated cardiac dysfunction and fibrotic remodeling induced by pressure overload

**FIGURE 7:**
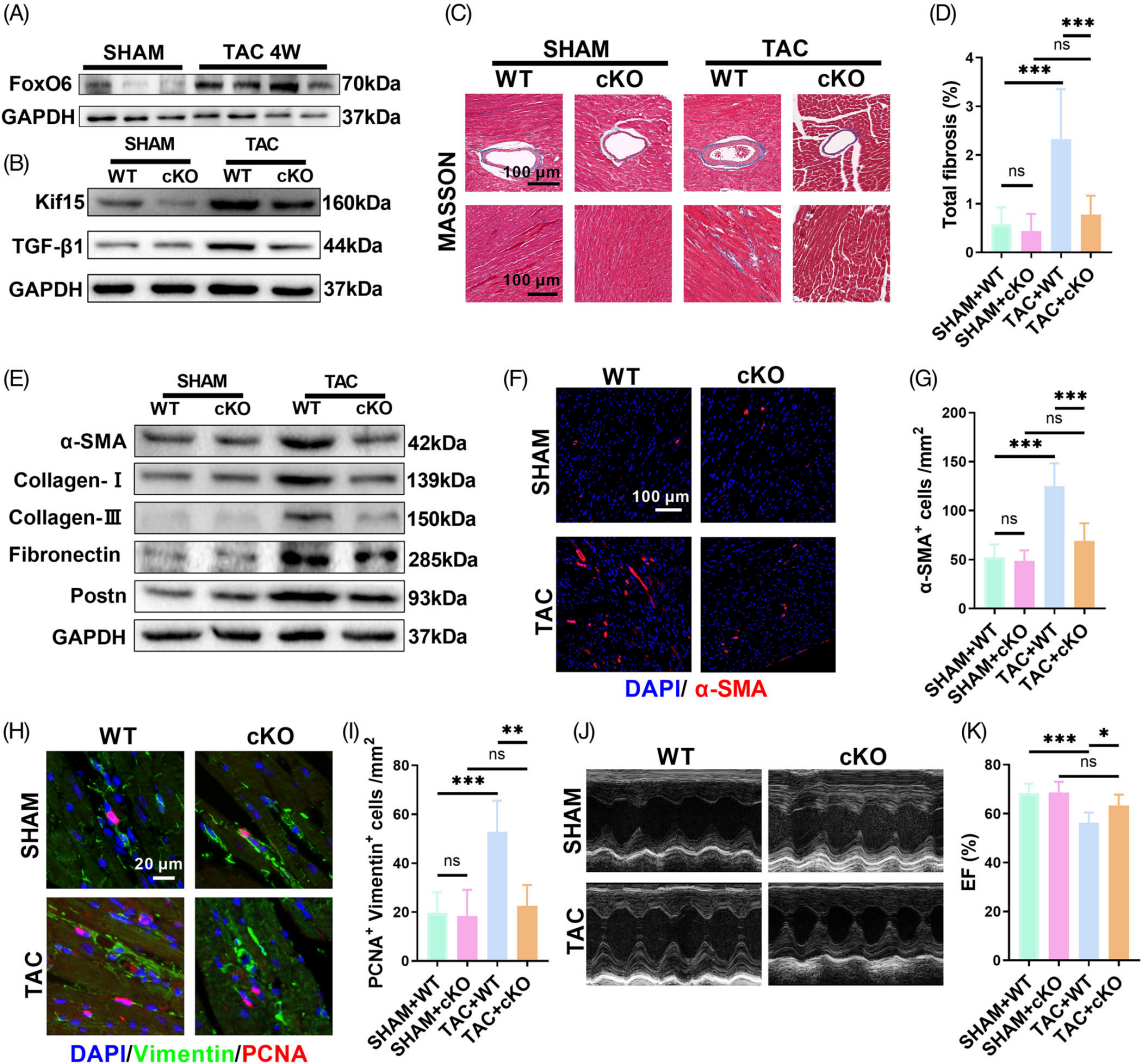
Myocardial‐specific knockdown of Forkhead box protein O6 (FoxO6) improved transverse aortic constriction (TAC)‐induced cardiac function impairment and myocardial fibrosis. (A) Typical western blots indicating FoxO6 in murine myocardium (*n* = 3−4 mice per group). (B) Typical western blots indicating Kif15 and transforming growth factor‐β1 (TGF‐β1) in murine myocardium (*n* = 6). (C) Typical images of murine heart sections (after 4 weeks of TAC) stained with Masson's trichrome stain, perivascular area, and interstitial area were, respectively, exhibited. (D) Degree of left ventricular (LV) fibrosis evidenced by collagen volume (*n* = 7). (E) Typical western blots indicating α‐SMA, collagen I, collagen III, fibronectin, and Postn in murine myocardium (*n* = 6). (F) Immunostaining of murine myocardium sections to exhibit the expression of α‐SMA (red). (G) Number of α‐SMA‐positive cells (*n* = 6). (H) Immunostaining of murine myocardium sections to exhibit the expression of vimentin (green) and proliferating cell nuclear antigen (PCNA) (red). (I) Number of vimentin‐ and PCNA‐positive cells (*n* = 6). (J) Echocardiography of mice. (K) Ejection fraction (EF)% determined via echocardiography (*n* = 8). Data were analyzed by one‐way analysis of variance (ANOVA). ^*^ *p* < 0.05, ^**^ *p* < 0.01, ^***^ *p* < 0.001, ^****^ *p* < 0.0001. Statistics are carried out as mean ± standard deviation (SD).

We developed a pressure overload mouse model by TAC surgery—a well‐recognized method for constructing murine HF models. Four weeks after surgery, the levels of FoxO6 were found to be significantly raised compared with those in the sham‐operated mice (Figure 7A). FoxO6 cKO reversed the upregulated expression of Kif15 and TGF‐β1 induced by pressure overload (Figure 7B). Cardiac fibrosis was significantly alleviated by FoxO6 deficiency, as evidenced by LV collagen volume (Figure 7C,D). The expression of fibrotic genes, such as *α‐sma*, *Col‐I*, *Col‐III*, *Fibronectin*, and *Postn*, was elevated in the hearts of the TAC mice, while FoxO6 deficiency reduced this effect (Figure 7E). Fibroblast differentiation and proliferation were measured by immunofluorescence staining. After TAC surgery, the number of α‐SMA‐positive cells increased, while the number remarkably decreased in TAC + FoxO6 cKO mouse hearts (Figure 7F,G). The number of PCNA‐positive and vimentin co‐positive cells increased significantly after TAC surgery, which was reversed by FoxO6 deficiency (Figure 7H,I).

After 4 weeks, the TAC surgery caused severe cardiac dysfunction, while FoxO6 cardiac‐specific deficiency resulted in discernible improvements in cardiac function (Figures 7J,K and S12A). The LV volume was higher in TAC‐treated FoxO6 cKO mice than in WT mice due to LV centripetal hypertrophy (Figure S12B). FoxO6 cKO, similar to the effects of Ang‐II, significantly attenuated cardiac hypertrophy after TAC surgery, as evidenced by the ventricular wall thickness measured by ultrasound and the cross‐sectional area of CMs (Figure S12C–I). There was also a significant decrease in the LW/BW and HW/BW ratios in FoxO6 cKO animals compared to WT animals following TAC surgery (Figure S12J,K).

We concluded that CM‐derived endogenous FoxO6 is involved in cardiac pathological remodeling when HF factors (such as Ang‐II and pressure overload) occur. FoxO6 cKO significantly inhibited cardiac hypertrophy and fibrotic remodeling and improved cardiac function, which was achieved by inhibiting the expression of Kif15. Reduced expression of Kif15 led to the downregulated secretion of TGF‐β1 by CMs, which attenuated the differentiation and proliferation of fibroblasts. The entire signaling pathway is mediated by FoxO6, which enhances communication between CMs and fibroblasts.

## 3 DISCUSSION

We identified elevated levels of FoxO6 expression in the myocardial tissue of mice exposed to HF risk factors that promote cardiac fibrosis and accelerate the progression of HF. This study appears to be the first to investigate FoxO6 expression and its function in HF specifically.

HF has a high incidence worldwide and a high 1‐year mortality rate, and is associated with reduced quality of life and overall health.^13^ When diseases such as hypertension and valvular heart disease occur, cardiac afterload increases, and compensatory hypertrophy of CMs initially enhances contractility.^14^ However, prolonged pressure overload results in decompensation, altered CM function, and the weakening of cardiac function. Pathological cardiac remodeling can increase myocardial cell necrosis or apoptosis, promote fibrosis remodeling, and eventually lead to HF.^15^ New treatments are urgently needed to reduce pathological cardiac remodeling. Our findings suggest a prospective therapeutic target for restraining pathological cardiac hypertrophy and HF.

The regulation of intercellular communication patterns is a significant contributor to the pathophysiological progression of various cardiac diseases.^16^, ^17^, ^18^ Our study showed that FoxO6 promoted the proliferation and differentiation of fibroblasts by regulating communication between CMs and fibroblasts, thereby accelerating cardiac fibrotic remodeling. FoxO6 achieved this effect by upregulating Kif15 expression, thereby increasing the secretion of TGF‐β1 in CMs. TGF‐β is a widely recognized stimulator of fibroblast activation.^19^

FoxO activity and expression are associated with the course of cardiovascular disease. FoxO expression and activation also play important roles in regulating diabetic cardiomyopathy, ischemic heart disease, and cardiac hypertrophy, among other heart‐related conditions.^20^ Numerous signaling mechanisms are involved in cardiac regulation. Notably, the functions of FoxO isoforms vary according to cardiac pathological responses, and their underlying mechanisms remain unclear.^21^

Of the four family members, FoxO6 is the most recent and least reported member and differs markedly from other FoxO isoforms in that it lacks the structural domain to receive nuclear transport signals and cannot perform insulin‐dependent nuclear transport.^22^ FoxO6 regulates hepatic glucolipid metabolism, which modulates very low‐density lipoprotein‐triglycerides (VLDL‐TG) production and gluconeogenesis.^22^, ^23^ In skeletal muscles, FoxO6 and peroxisome proliferator activating receptor gamma coactivator 1 (PGC‐1α) form a feedback loop in which they interact to regulate oxidative metabolism in response to exercise.^12^ Although FoxO6 is abundantly expressed in myocytes, its role in heart and cardiac diseases remains unclear. It has been shown that downregulation of FoxO6 expression reduces oxidative stress injury in hypoxic CMs, although further in vivo verification is required.^10^

The pathological cardiac remodeling caused by neurohumoral activation (e.g., through Ang‐II) is significantly associated with the development of HF. Adrenergic receptor blockers, angiotensin‐converting enzyme inhibitors, and angiotensin receptor blockers are widely used to reduce LV mass and slow HF progression, but they do not completely inhibit the progression of HF.^24^ Here, Ang‐II administration led to cardiac fibrosis, hypertrophy, and reduced cardiac function, together with increasing the levels of FoxO6. The knockout and overexpression of FoxO6 in the heart attenuated and exacerbated cardiac hypertrophy and fibrosis, which was directly associated with myocardial contractile function.

Several genes are closely related to cardiac fibrosis. Ang‐II affects the expression patterns of these marker genes, which regulate processes related to fibrosis, including fibroblast proliferation, differentiation, collagen synthesis, secretion, and ECM reconstruction.^25^, ^26^ Fibrosis‐associated gene expression was increased after Ang‐II stimulation, while FoxO6 deficiency and overexpression attenuated and exacerbated this effect, respectively. We found that FoxO6 promoted the proliferation and differentiation of fibroblasts, representing activation of fibroblasts.^27^

We explored the mechanism of action of FoxO6 in two ways. First, we detected recognized signaling pathways involved in fibroblast function, such as TGF‐β, integrins, and inflammatory signaling factors, among others.^25^ FoxO6 regulated TGF‐β1 expression in the hearts of Ang‐II‐treated mice. TGF‐βs are the most widely studied molecules in pathological fibrosis, with TGF‐β1 being the main signaling pathway.^28^ These results demonstrate the noteworthy role of FoxO6 in developing cardiac fibrosis. Second, we evaluated the downstream molecules related to FoxO6 by RNA sequencing of mouse heart tissues from the NULL + Ang‐II and FoxO6 + Ang‐II groups.^29^ FoxO6 overexpression mainly induced the expression of genes associated with fibrosis regulation, such as collagen formation, ECM organization, and pathways mediating cardiac fibrosis. We identified Kif15 as the most significant DEG.

Kinesins are a class of motor‐like molecules that move unidirectionally along intracellular microtubules, play various vital roles in intracellular transport and cell division, and have been widely studied in multiple cancers.^30^ Kif15 has been linked with tumor progression in many cancers, including gastric and breast cancers, by promoting cell proliferation and migration.^31^, ^32^ A new clinical study reported that a Kif15 mutation contributes to idiopathic pulmonary fibrosis, which is closely associated with the function of Kif15 in regulating cell proliferation.^33^ However, the specific mechanism by which Kif15 regulates fibrosis has not yet been reported. Our results suggest that cardiac fibrosis is regulated by both Kif15 and TGF‐β1 signaling.

In Ang‐II‐treated adeno‐associated virus 9 (AAV9)‐FoxO6‐transfected WT mice treated with Kif‐15‐IN‐1, we found for the first time that Kif15 mediated the pro‐fibrosis effect of FoxO6 and inhibition of Kif15 blunted the upregulation of TGF‐β1 expression by FoxO6. However, we found that Ang‐II only elevated FoxO6 expression in CMs rather than in other cells from myocardial tissue. In vitro experiments verified that FoxO6 indeed regulated the expression of Kif15 and TGF‐β1 in NRCMs but did affect Ang‐II‐stimulated CM hypertrophy, whereas, in TGF‐β‐treated fibroblasts, changes in FoxO6 did not affect the degree of differentiation. We hypothesized that FoxO6 activates fibroblasts by regulating the expression of Kif15 and secretion of TGF‐β1 in CMs.

The pathophysiological processes of all heart diseases, including HF, occur through intercellular interactions.^34^ As for cardiac fibrosis, several studies have reported mechanisms related to intercellular myocardial communication. Many molecules—including miR‐208a, miR‐133, and miR‐378—secreted from CMs regulate fibroblasts.^35^, ^36^, ^37^ Additionally, the endothelial cell‐derived transcription factor Forkhead box protein P1 (Foxp1) promotes the expression and secretion of TGF‐β1, thus promoting the progression of myocardial fibrosis.^19^ Therefore, we cultured fibroblasts in an NRCM culture medium (after treatment) and found for the first time that FoxO6 promoted the secretion of TGF‐β1 by upregulating Kif15, thereby activating fibroblasts. FoxO6, as a transcriptional regulator, directly upregulates the expression of Kif15 in CMs, as evidenced by genomics detection, western blotting, and immunofluorescence staining.

Kif15 mediates the pro‐hypertrophic role of FoxO6 in CM, whereas TGF‐β1 derived from CMs is secreted to extracellularly under activated Kif15 and activates the proliferation and differentiation of fibroblasts. TAC surgery—a classic pressure‐overload HF model—is a direct method of constricting the aortic arch.^38^ Following TAC surgery, the knockout of FoxO6 expression in the myocardium played a significant role in anti‐hypertrophy and improved cardiac function, further confirming our conclusion.

For the first time, we have elucidated that FoxO6 in CMs promotes pathological cardiac fibrosis induced by pressure overload largely by activating Kif15, which contributes to TGF‐β1 secretion. Our study identified a potential signaling pathway for CMs to communicate with fibroblasts during HF and a potential therapeutic approach for patients with chronic HF through downregulating FoxO6 expression in myocardial tissue. In future studies, we will further explore the role and significance of FoxO6 in the progression of heart disease. First, we will continue this study to clarify further the regulatory effects of FoxO6 on gene expression, metabolism, and lifespan in the CMs of mice with afterload‐induced HF model. Second, to explore the role and significance of FoxO6 in cardiac pathophysiological regulation, we will attempt to construct various mouse cardiac disease models, including myocardial infarction, ischemia–reperfusion injury, coronary atherosclerosis, and diabetic cardiomyopathy models with FoxO6 overexpression or knockout. FoxO6 is expected to be an important intervention target for heart disease and even HF.

## 4 MATERIALS AND METHODS

### 4.1 Experimental animals

FoxO6 cKO male mice were generated at Nanjing Biomedical Research Institute of Nanjing University. C57BL/6 mice (male, 20−25 g, 8−10 weeks old) were purchased from Experimental Animal Center of the Fourth Military Medical University. The animals were maintained under standard conditions, namely, between 22°C and 24°C, 12‐h/12‐h light/dark cycle (lights switched on at 06:00), and free access to standard mouse chow and water, with 10‒12 mice per cage. All protocols, including the raising and use of mice, were conducted following the Guide for the Care and Use of Laboratory Animals of the Chinese Animal Welfare Committee. Study protocol was approved by the Fourth Military Medical University Committee on Animal Care. Mice were subcutaneously infused with saline (control) or Ang‐II (1.3 mg/kg/day) for 4 weeks using an osmotic mini‐pump to cause cardiac hypertrophy.

### 4.2 Animal experimental design and drug administration

In experiment 1, mice were divided into eight groups for 4‐week treatments: (1) WT + saline (WT mice receiving saline); (2) WT + Ang‐II (WT mice receiving Ang‐II); (3) FoxO6 cKO + saline (FoxO6 cKO mice receiving saline); (4) FoxO6 cKO + Ang‐II (FoxO6 cKO mice receiving Ang‐II); (5) NULL + saline (WT mice receiving an intramyocardial injection of AAV9‐NULL and then saline); (6) NULL + Ang‐II (WT mice receiving an intramyocardial injection of AAV9‐NULL and then Ang‐II); (7) FoxO6 + saline (WT mice receiving an intramyocardial injection of AAV9‐FoxO6 and then saline); and (8) FoxO6 + Ang‐II (WT mice receiving an intramyocardial injection of AAV9‐FoxO6 and then Ang‐II).

In experiment 2, mice were divided into four groups: (1) WT + SHAM (WT mice receiving sham surgery); (2) WT + TAC (WT mice receiving TAC surgery); (3) FoxO6 cKO + SHAM (FoxO6 cKO mice receiving sham surgery); and (4) FoxO6 cKO + TAC (FoxO6 cKO mice receiving TAC surgery).

In experiment 3, mice were divided into four groups for 4‐week treatments: (1) NULL + Ang‐II (WT mice receiving an intramyocardial injection of AAV9‐NULL and then Ang‐II); (2) FoxO6 + Ang‐II (WT mice receiving an intramyocardial injection of AAV9‐FoxO6 and then Ang‐II); (3) Kif15‐IN‐1 + Ang‐II (WT mice receiving an intramyocardial injection of AAV9‐NULL and then Ang‐II with Kif15‐IN‐1 [a Kif15 inhibitor; 0.05 mg diluted in 0.5% dimethyl sulfoxide, intraperitoneally injected every 2 days]); and (4) FoxO6 + Kif15‐IN‐1 + Ang‐II (WT mice receiving an intramyocardial injection of AAV9‐FoxO6 and then Ang‐II with Kif15‐IN‐1). Instead of Kif15‐IN‐1, groups (1) and (2) were administered an equivalent dose of dimethyl sulfoxide every 2 days.

### 4.3 Echocardiography

The echocardiographer was blinded to both the surgical and experimental protocols. The mice were anesthetized as described above and placed on a 42°C thermostatic plate with their body temperatures maintained at approximately 38°C. The isoflurane concentration was adjusted to maintain a heart rate of 400−500 beats per minute. Transthoracic ultrasonography was conducted using echocardiograph VisualSonics 770 (FUJIFILM VisualSonics). A 30‐MHz transducer was used to record the parasternal long and short axes of the left ventricle. The indicators of cardiac hypertrophy were the LV wall thickness and LV volume. The indicators of cardiac function were LVEF and LVFS. Measurements and calculations were performed using Vevo Lab 3.1.0 software (FUJIFILM VisualSonics).

### 4.4 RNA extraction, library construction, and sequencing

Total RNA of myocardium was extracted via a TRIzol reagent kit (Invitrogen), following the provided directions. An Agilent 2100 Bioanalyzer (Agilent Technologies) and RNase‐free agarose gel electrophoresis were used to evaluate the quality of the RNA. Eukaryotic mRNA was enriched by oligo (dT) beads and was then fragmented using a fragmentation buffer. Reverse‐transcription of the RNA to cDNA was performed with random primers. Then, second‐strand cDNA was synthesized with the assistance of DNA polymerase I, RNase H, dNTP, and buffer. The cDNA fragments were purified using a QiaQuick PCR extraction kit (Qiagen). Then the cDNA was end‐repaired, treated with poly(A), and ligated to Illumina sequencing adapters. We used agarose gel electrophoresis to size‐selected the ligation products. And the selected products were PCR‐amplified and sequenced using an Illumina NovaSeq 6000 (Gene Denovo Biotechnology). DEGs were identified by comparing RNA expression between groups using a threshold of *p* < 0.05 and an absolute fold change ≥1.5. Gene annotations and pathway enrichment were analyzed using the GO and Kyoto Encyclopedia of Genes and Genomes (KEGG) (http://www.genome.ad.jp/kegg) databases, respectively. The calculated *p*‐value was false discovery rate (FDR)‐corrected with a threshold ≤0.05.

### 4.5 Histology

Myocardium tissue embedded in paraffin was cut into sections and then stained with HE or Masson's trichrome stain. The CM cross‐sectional areas and collagen deposition were assessed as described previously.^39^ The tissue sections were evaluated with an FV1000 digital scanning imaging system (Olympus). We used ImageJ software (NIH) to analyze cross‐sectional areas and the proportion of fibrosis.

### 4.6 Immunofluorescence

Immunofluorescence staining was conducted using a previously published method.^40^ To analyze the levels of FoxO6, tissue sections were first permeabilized with 0.05% Triton X‐100 for 10 min, followed by three washes in phosphate buffered saline (PBS) and incubation with 1% bovine serum albumin (BSA) for 1 h at 25°C. The sections were then probed with rabbit anti‐FoxO6 antibody (see Section 4.8, 1:50 dilution) for 10−12 h at 4°C. Then, the sections were washed with PBS and incubated with goat anti‐rabbit or anti‐mouse secondary antibodies conjugated to Alexa Fluor Cy3. 4',6‐diamidino‐2‐phenylindole (DAPI) was used to counterstain the nuclei, and the FoxO6 levels were determined by the intensity of fluorescence.

The surface areas of the CMs and the proliferation and differentiation of CFs were assessed using immunofluorescence. The treated cells in confocal dishes containing NRCMs were rinsed three times with PBS and 4% paraformaldehyde was used to fix cell morphology. After three more washes in PBS and permeabilization with 0.05% Triton X‐100 for 10 min, followed by further washing, the cells were incubated with 1% BSA for 30 min at approximately 27°C followed by incubation with α‐actinin (Sigma A7732, Sigma–Aldrich) for 10−12 h at 4°C. The cells were then incubated with horseradish peroxidase (HRP)‐conjugated secondary antibody at 37°C for 2 h, washed again, and the nuclei were counterstained with DAPI. Cells were examined under confocal microscopy, analyzing at least 50 cells per treatment group, and CM surface areas were calculated using ImageJ software.

The CMs or CFs were fixed, permeabilized, and blocked as described above before incubation with primary antibodies in PBS overnight at 4°C. The cells were then treated with a fluorescent secondary antibody in PBS for 2 h at room temperature and, after counterstaining the nuclei with DAPI, were analyzed under confocal microscopy.

### 4.7 qRT‐PCR

Four weeks after surgery (both TAC and sham), eight mice were selected from each group, weighed, and sacrificed by exsanguination from the carotid artery. The hearts were collected, flushed using PBS, and weighed. HW/BW was then determined. A small tissue sample was excised from left ventricle for extraction of total RNA using TRIzol (TIANGEN) and reverse‐transcribed to cDNA using a SuperScript First‐Strand Synthesis System (Invitrogen). qPCR was performed on a CFX96 real‐time PCR system C1000 Thermal Cycler (Bio‐Rad Laboratories) using *Gapdh* for normalization. The primers used were synthesized by GenScript Biotechnology (Table S1).

### 4.8 Western blotting

The protein contents of the left ventricles and NRCMs were extracted in radio immunoprecipitation assay (RIPA) lysis buffer. Protein concentrations were measured by bicinchoninic acid assay. Proteins were separated by 10% sodium dodecyl sulfate polyacrylamide gel electrophoresis (SDS‐PAGE). Then, the blots were transferred to polyvinylidene difluoride membranes (Millipore). After blocking the membranes in 5% fat‐free milk powder in Tris‐buffered saline tween‐20 (TBST) (150 mM NaCl, 50 mM Tris [pH 7.5], and 0.1% Tween‐20) for 2−3 h at 20°C−25°C, we cut them into bands according to their molecular weights and incubated with their appropriate primary antibodies for 12 h at 4°C before incubation with the appropriate HRP‐conjugated secondary antibody and visualization with enhanced chemiluminescence (ECL). The blots were scanned with ChemiDoc XRS (Bio‐Rad Laboratories), and the gray values of the bands were analyzed using Image Lab 2.0 (Genmall Biotechnology). Primary antibodies against TGF‐β1, α‐SMA, collagen I, collagen III, fibronectin, Postn, ANP, and Kif15 were purchased from Abcam, while the anti‐α‐actinin antibody was from Sigma–Aldrich, anti‐β‐MHC and Ki67 antibodies were from Santa Cruz Biotechnology and the anti‐FoxO6 antibody was from Proteintech. GAPDH was used as the internal control.

### 4.9 Statistical analysis

The recorded data were processed and analyzed using GraphPad Prism 7.0 (GraphPad Software). The data are presented as means ± standard deviation. The differences between two groups were assessed using *t*‐tests, while the differences between multiple groups were analyzed via a one‐way analysis of variance. We considered *p* < 0.05 to be statistically significant.

## AUTHOR CONTRIBUTIONS

All authors revised and approved the final paper. W.Y., D.Y., and Y.S. designed the study and modified the manuscript. J.L., W.D., and Z.J. were responsible for reviewing the data. B.Z., Y.T., L.S., Y.Z., J.C., and Y.S. implemented the experiments and collected data. Y.L., M.Z., and Y.Z. analyzed the data. B.Z., Y.T., L.S., and Y.Z. drafted this paper. All authors have read and approved the final manuscript.

## CONFLICT OF INTEREST STATEMENT

The authors declare they have no conflicts of interest.

## ETHICS STATEMENT

All institutional and national guidelines for the care and use of animals were followed.

All experiments comply with the ARRIVE guidelines. All procedures, including the raising and use of mice, were performed according to the Guide for the Care and Use of Laboratory Animals of the Chinese Animal Welfare Committee, and the Fourth Military Medical University Committee on Animal Care approved the study protocol (approval number: IACUC‐20201289).

## Supporting information

Supporting Information

## Data Availability

The datasets used and analyzed during the current study are available from the corresponding author or the first author upon reasonable request.
