# Supplementary material for: Forkhead box O6 (FoxO6) promotes cardiac pathological remodeling and dysfunction by activating Kif15–TGF‐β1 under aggravated afterload
Source: MedComm (2020). 2023 Oct 3;4(5):e383. doi: 10.1002/mco2.383 (PMC10547936; doi:10.1002/mco2.383)
Supplement: Supplementary file 1 — Supporting Information [file MCO2-4-e383-s001.docx]

**Forkhead Box O6 (FoxO6) promotes cardiac pathological remodeling and dysfunction by activating Kif15-TGF-β1 under aggravated afterload**

**Running title:** FoxO6 promotes afterload induced cardiac fibrosis

Bing Zhang^1#^, Lei Shi^1#^, Yanzhen Tan^1#^, Yenong Zhou^1#^, Jun Cui^1^, Yujie Song^1^, Yingying Liu^1^, Miao Zhang^2^, Weixun Duan^1^, Zhenxiao Jin^1^, Jincheng Liu^1^, Dinghua Yi^1^, Yang Sun^2*^, Wei Yi^1*^

^1^ Department of Cardiovascular Surgery, Xijing Hospital, The Fourth Military Medical University, 127 Changle West Road, Xi’an 710032, China

^2^ Department of Geriatrics, Xijing Hospital, The Fourth Military Medical University, 127 Changle West Road, Xi’an 710032, China

Bing Zhang, Lei Shi, Yanzhen Tan, and Yenong Zhou contributed equally to this work.

Corresponding authors:

Wei Yi ([yiwei@fmmu.edu.cn](mailto:yiwei@fmmu.edu.cn)). Department of Cardiovascular Surgery, Xijing Hospital, The Fourth Military Medical University, 127 Changle West Road, Xi’an 710032, China.

Yang Sun ([dr_yangsun@163.com](mailto:dr_yangsun@163.com)). Department of Geriatrics, Xijing Hospital, The Fourth Military Medical University, 127 Changle West Road, Xi’an 710032, China

**Supplementary methods**

**Adeno-associated virus 9 (AAV9) preparation and injection**

Mouse-FoxO6-overexpressing recombinant AAV9 was constructed by Shanghai GeneChem (Shanghai, China). WT mice, five to seven weeks old, were anesthetized with a 2% (v/v) isoflurane-pure oxygen mixture in an induction chamber (0.5–1.0 L/min) and injected intramyocardially as described previously^1^. An oblique incision, approximately 0.5 cm in length, was made to the left of the sternum between the fourth and fifth costal margins, and the heart was pressed against the right wall of the chest. AAV9 (total volume of 25 μL with 5 × 10^8^ drips) was then injected intramuscularly into the front, back, and side of the left ventricle. The control mice received vehicle only (saline, LV-NULL). After repositioning the heart within the chest cavity, the chest was sutured. Sham and TAC procedures were conducted after three weeks.

**TAC**

TAC was used to establish the mouse model of chronic cardiac hypertrophy, following a published protocol^2^. Specifically, the mice were anesthetized as described above, followed by oral ventilation using a 20-gauge tube (Minivent Type 845, Hugo Sachs Electronik, March, Germany) at 100–120 mL/min (0.15-mL tidal volume). The thorax was opened in the midline at the second intercostal space. Constriction of the transverse aorta was performed by tying a 7-0 silk-suture ligature against a 27-gauge needle between the carotid arteries, followed by the rapid removal of the needle. The sham procedure was the same apart from the aortic ligation. The chest was closed using a 6-0 silk suture with the reattachment of the skin with a 5-0 silk suture. The mice were warmed on a 38 °C constant-temperature plate and monitored continuously until they regained consciousness.

**Isolation and culture of cardiomyocytes (CMs) and cardiac fibroblasts (CFs)**

Cells were isolated enzymatically with collagenase type II (Worthington) in a Langendorff perfusion system, following a previously published protocol ^3^. Specifically, the adult mice were anesthetized by intraperitoneal administration of heparin and ketamine/midazolam and euthanized using cervical dislocation. The heart was removed, washed briefly to eliminate the excess fat and blood, and placed in a heart perfusion apparatus with a VWR heating pump (Radnoti, Monrovia, CA, USA). The heart was then perfused in the Langendorff mode with Ca^2+^-free Krebs–Henseleit-based perfusion buffer at 37 °C (pH 7.4; filtered through at 0.40-μm filter). The buffer recipe included 120 mmol/L NaCl, 15 mmol/L KCl, 0.6 mmol/L Na_2_HPO_4_, 0.6 mmol/L KH_2_PO_4_, 1.2 mmol/L MgSO_4_, 10 mmol/L HEPES, 10 mmol/L creatine monohydrate, 30 mmol/L taurine, 5.6 mmol/L D-glucose, 4.6 mmol/L NaHCO_3_, and 10 mmol/L BDM. Perfusion was conducted with this buffer for 3 min, then with calcium-free digestion buffer (perfusion buffer containing 1.3 mg/mL collagenase II) for 3 min and digestion buffer containing 28 nmol/L CaCl_2_ for 10 min. The heart was then removed from the cannulating needle and placed in 2.5 mL calcium-containing digestion buffer and 5 mL of stopping buffer (perfusion buffer containing 10% fetal bovine serum [FBS] and 12.5 nmol/L CaCl_2_). After removing the right ventricle and atria, the left ventricle was placed in a petri dish, and the tissue dissociated with forceps before adding 10 mL of stopping buffer and filtrating through a 200-μm sieve into a 50-mL conical tube. The tissue was incubated at 37 °C for 10 min and a further 10 min at room temperature to allow sedimentation. After centrifuging the supernatant (1000 × *g*, 10 min), the primary CFs were collected and grown in Dulbecco’s Modified Eagle Medium (DMEM) with 10% FBS and 2% penicillin/streptomycin (P/S). The precipitate containing the CMs was then resuspended in 10 mL stopping buffer, and the buffer calcium concentration was raised step by step at 2-min intervals to 112.5 nmol/L, 512.5 nmol/L, and 1.4 μmol/L. The appearance of the CMs was evaluated, and cells, where over 60% were rod-shaped, were used for further study. The CMs were centrifuged (500 × *g,* 1 min), resuspended in plating medium (perfusion buffer with 2.5% FBS, 2% P/S, and 1.4 μmol/L CaCl_2_), seeded in dishes pre-coated with 9–10 μg/mL laminin, and incubated at 37 °C for 2–4 h. After the removal of dead cells with two washes of phosphate-buffered saline (PBS), the CMs were incubated in CM culture medium (DMEM with 0.02% bovine serum albumin [BSA], 10 mmol/L HEPES, 4 mmol/L NaHCO_3_, 10 mmol/L creatine, 0.5% insulin-selenium-transferrin, and 10 mmol/L BDM, pH 7.4) for 30‒60 min before use. For the chronic studies, such as the CM hypertrophy, the cells were grown in the medium containing 10 µM blebbistatin (a myosin II ATPase inhibitor) to prevent contraction and enhance myocyte survival ^3^.

**NRCM culture and treatment**

Newly born Sprague Dawley (SD) rats were purchased from the Experimental Animal Center of the Air Force Medical University. NRCMs were isolated and cultured following a published protocol ^1^. After harvesting the heart, the tissue was incubated in 1% collagenase I (V900891; Sigma-Aldrich, St. Louis, MO, USA) in PBS. The isolated NRCMs were seeded at densities of 5 × 10^5^ cells/mL and grown in DMEM/F-12 (Gibco, Waltham, MA, USA) containing 10% FBS, 100 U/mL penicillin, 100 U/mL streptomycin, and 0.1 mM bromodeoxyuridine (BrdU) for 48 h at 37 °C ND 5% CO_2_. The cells were then treated with 1 μmol/L Ang-II for 48 h to induce CM hypertrophy ^4^. Hypertrophy induction was monitored by α-actinin to measure the increases in the cell surface area and expression of specific markers: myosin heavy chain β (β-MHC) and atrial natriuretic peptide (ANP). To induce the overexpression of FoxO6, the cells were infected with adenovirus (Ad)-FoxO6 or Ad-null for 4 h at a multiplicity of infection (MOI) of 60 (virus dose was 3 × 10^7^ pfu/mL). For the FoxO6 and Kif15 knockdown, the NRCMs were transfected with FoxO6 siRNA and Kif15 siRNA using a Lipofectamine 3000 system (Thermo Fisher Scientific, Waltham, MA, USA) ^5^. The cells were treated with Ang-II 24 h later. The knockdown efficiency was confirmed by western blotting.

**Fibroblast culture and treatment**

Fibroblasts were plated onto a 35-mm dish for several hours to allow for attachment. After three washes with PBS, the cells were grown in DMEM + 10% (v/v) FBS + 2% (v/v) P/S for two days. This was followed by culture in serum-free medium for 24 h, after which the medium was replaced with NRCM media from the different groups.

**Reference**

1. Zhang B, Zhang P, Tan Y, et al. C1q-TNF-related protein-3 attenuates pressure overload-induced cardiac hypertrophy by suppressing the p38/CREB pathway and p38-induced ER stress. *Cell Death Dis*. 2019;10(7):520.

2. Wang J, Gao E, Chan TO, et al. Induced overexpression of Na(+)/Ca(2+) exchanger does not aggravate myocardial dysfunction induced by transverse aortic constriction. *J Card Fail*. 2013;19(1):60-70.

3. Knight WE, Chen S, Zhang Y, et al. PDE1C deficiency antagonizes pathological cardiac remodeling and dysfunction. *Proc Natl Acad Sci U S A*. 2016;113(45):E7116-e7125.

4. Okabe K, Matsushima S, Ikeda S, et al. DPP (Dipeptidyl Peptidase)-4 Inhibitor Attenuates Ang II (Angiotensin II)-Induced Cardiac Hypertrophy via GLP (Glucagon-Like Peptide)-1-Dependent Suppression of Nox (Nicotinamide Adenine Dinucleotide Phosphate Oxidase) 4-HDAC (Histone Deacetylase) 4 Pathway. *Hypertension*. 2020;75(4):991-1001.

5. Zhai M, Liu Z, Zhang B, et al. Melatonin protects against the pathological cardiac hypertrophy induced by transverse aortic constriction through activating PGC-1beta: In vivo and in vitro studies. *Journal of pineal research*. 2017;63(3)

**Supplementary figures**


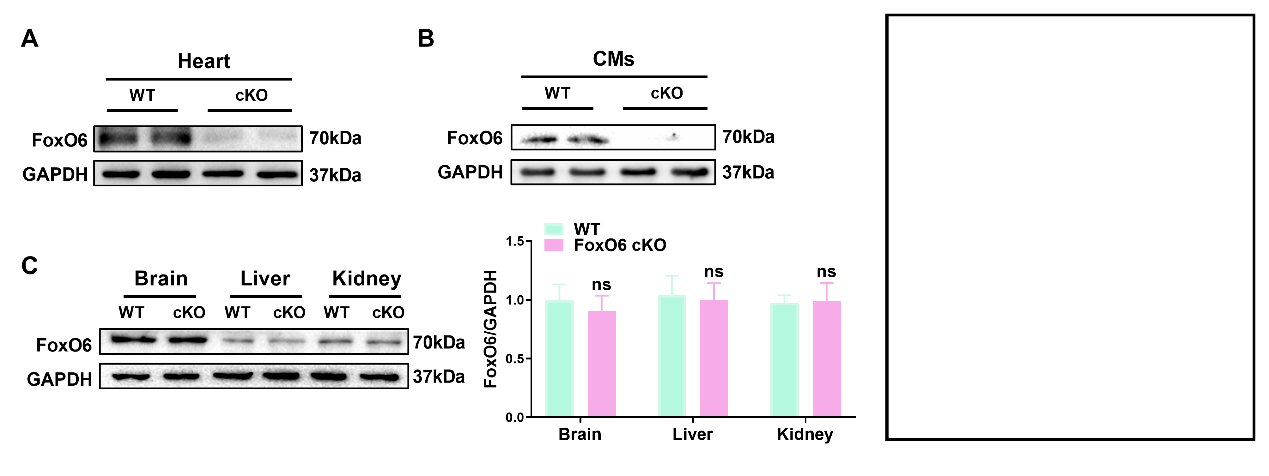


**Figure S1. a** Typical western blot indicating FoxO6 in murine myocardium. **b** Typical western blots indicating FoxO6 in cardiomyocytes separated from murine hearts. **c** Cardiac protein expression levels of FoxO6 in different tissues of mice (n = 4). Data were analyzed by *t*-test. ^ns^p > 0.05 vs. WT group. Statistics are carried out as mean ± standard deviation (SD).


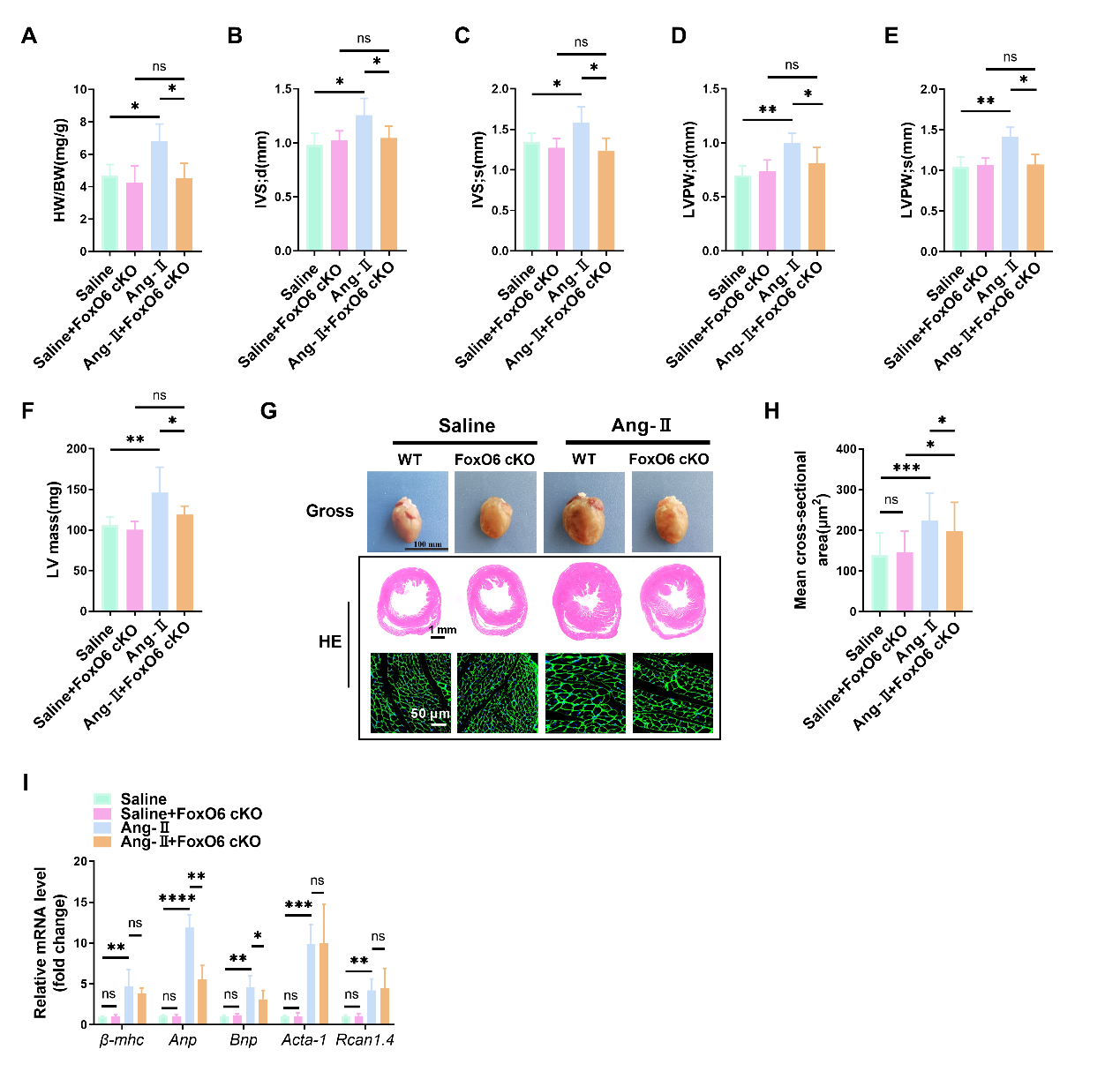


**Figure S2.** (**A**) HW/BW ratio of mice (n = 5 mice per group). (**B–F**) IVSd, IVSs, LVPWd, LVPWs, and LV vol, respectively, determined via echocardiography (n = 8–13). (**G**) Gross anterior view of murine hearts and typical images of sections stained with hematoxylin and eosin (HE) and wheat germ agglutinin (WGA) (**H**) Analysis of CMs cross-sectional area (n ≥ 100 cells/group). (**I**) Cardiac mRNA expression levels of genes encoding hypertrophic markers β-MHC, ANP, BNP, Acta-1, and Rcan1.4 (n = 6). Data were analyzed by one-way ANOVA. *p < 0.05, **p < 0.01, ***p < 0.001, ****p < 0.0001. Statistics are carried out as mean ± standard deviation (SD).


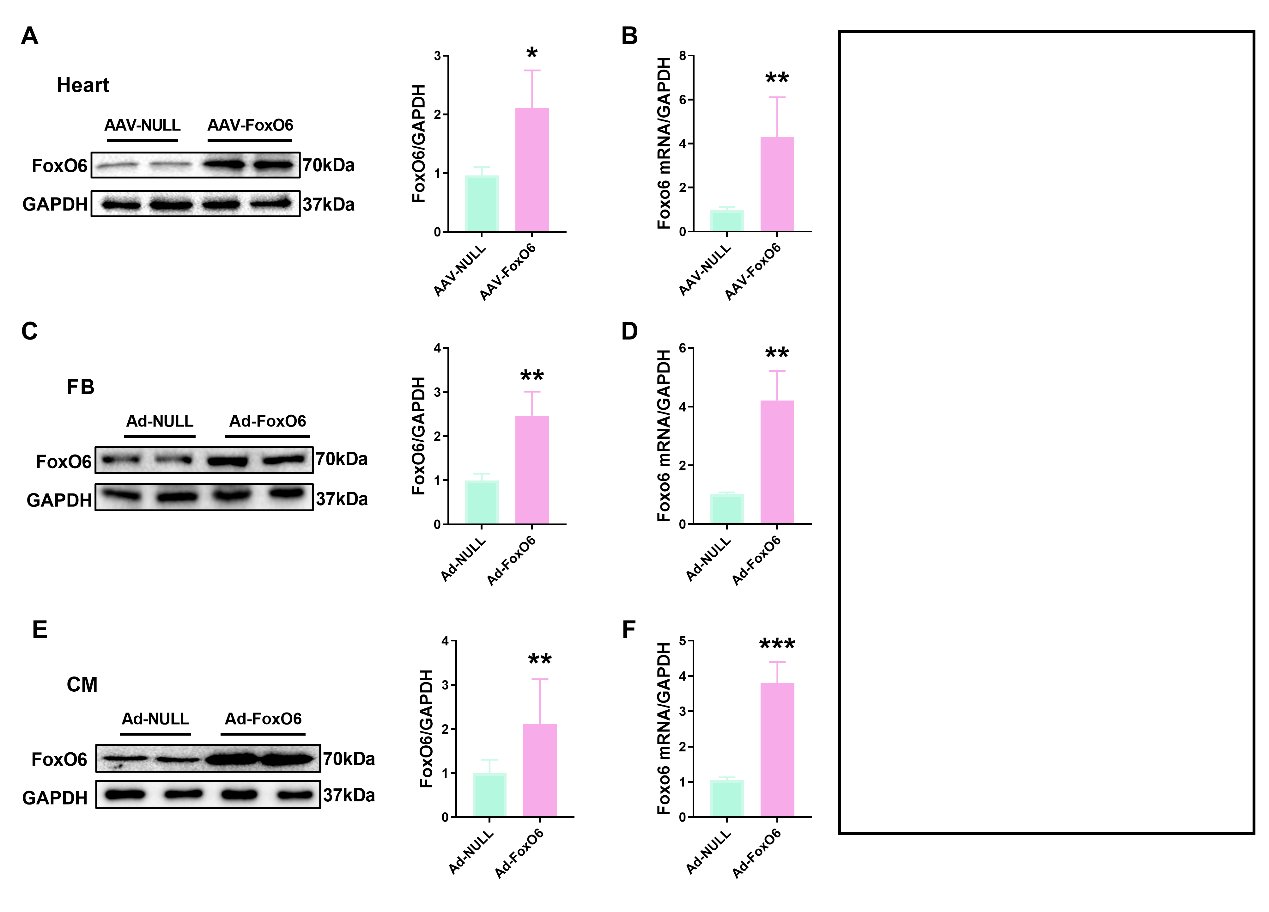


**Figure S3.** (**A**) Cardiac protein expression level of FoxO6 in heart tissues of mice from indicated groups (n = 4 mice per group). (**B**) Cardiac mRNA expression level of gene encoding FoxO6 in each group (n = 4). (**C**) FB protein expression level of of FoxO6 (n = 4 samples per group). (**D**) FB mRNA expression level of gene encoding FoxO6 in each group (n = 4). (**E**) CM protein expression level of FoxO6 (n = 4). (**F**) CM mRNA expression level of gene encoding FoxO6 in each group (n = 4). Data were analyzed by *t*-test. *p < 0.05, **p < 0.01, ***p < 0.001. Statistics are carried out as mean ± standard deviation (SD).


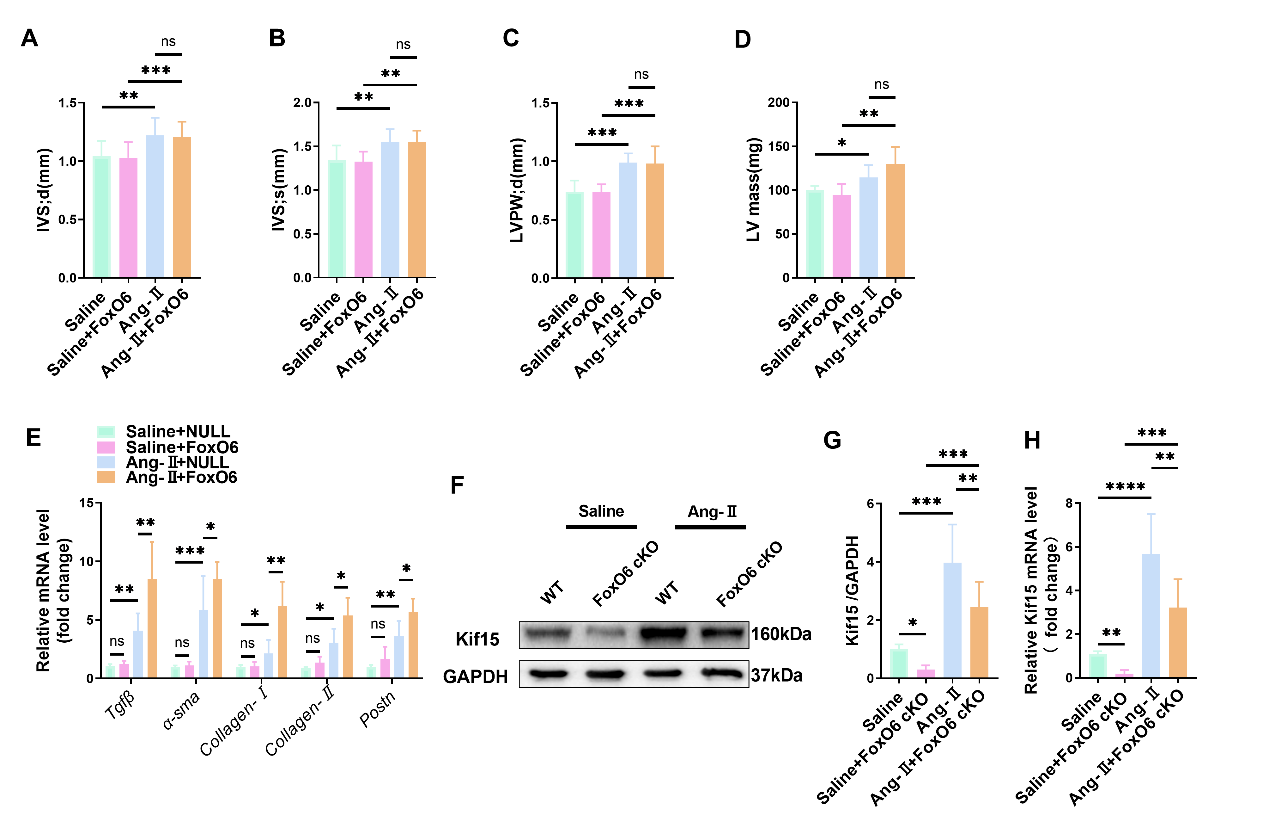


**Figure S4. (A–D**) IVSd, IVSs, LVPWd, and LV mass, respectively, determined by echocardiography. (**E**) Cardiac mRNA expression levels of genes encoding fibrotic markers TGF-β1, α-SMA, collagen-Ⅰ, collagen-III, and Postn (n = 6 mice per group). (**F**) Typical western blot indicating Kif15 in murine myocardium. (**G**) Cardiac protein expression level of Kif15 in murine myocardium (n = 6). (**H**) Cardiac mRNA expression level of gene encoding Kif15 in each group (n = 6). Data were analyzed by one-way ANOVA. *p < 0.05, **p < 0.01, ***p < 0.001, ****p < 0.0001. Statistics are carried out as mean ± standard deviation (SD).


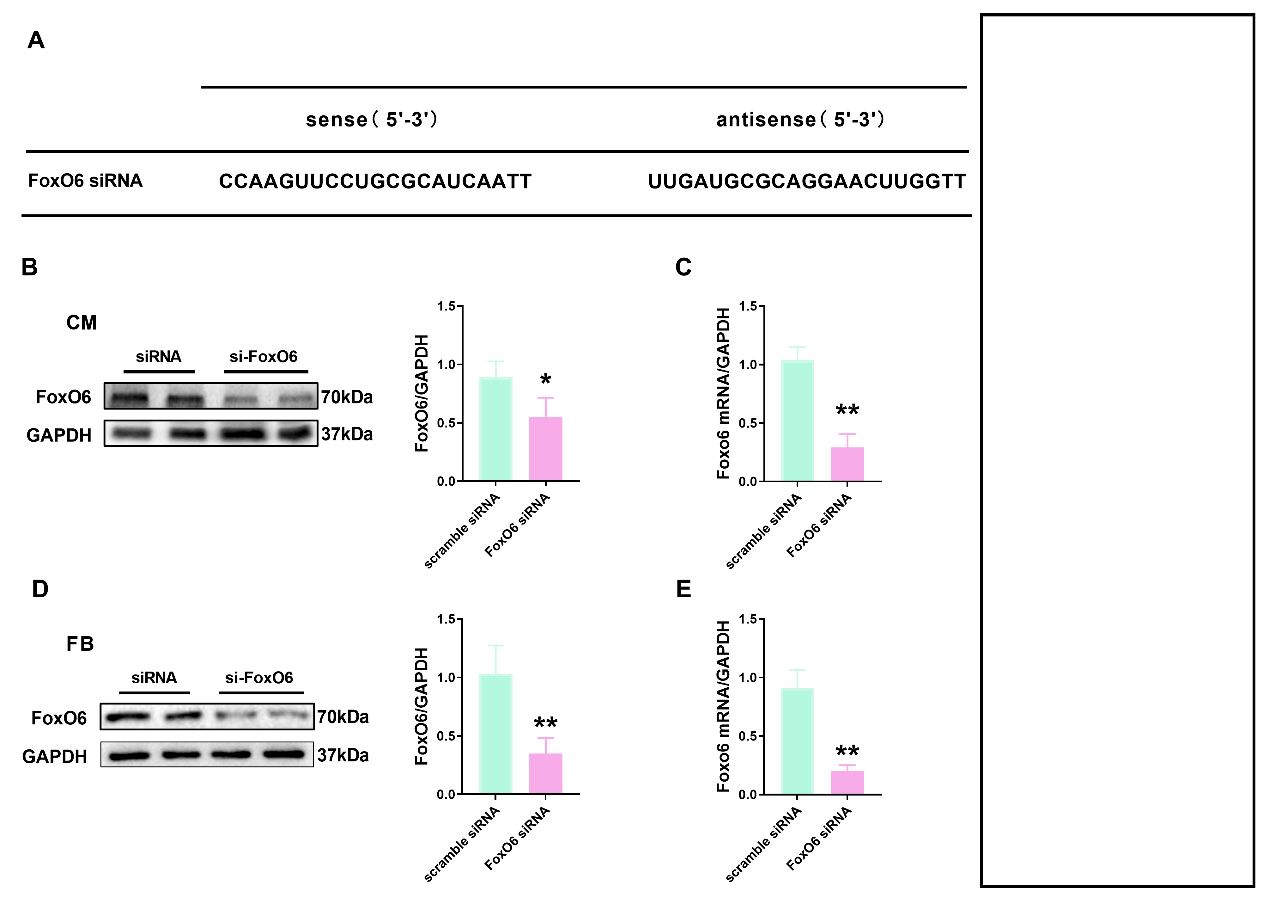


**Figure S5.** (**A**) FoxO6 siRNA with most significant effect of intracellular FoxO6 expression knockdown. (**B**) CM protein expression level of FoxO6 (n = 4 samples per group). (**C**) CM mRNA expression level of gene encoding FoxO6 (n = 4). (**D**) FB protein expression level of of FoxO6 (n = 4 samples per group). (**E**) FB mRNA expression level of gene encoding FoxO6 (n = 4). Data were analyzed by *t*-test. *p < 0.05, **p < 0.01. Statistics are carried out as mean ± standard deviation (SD).


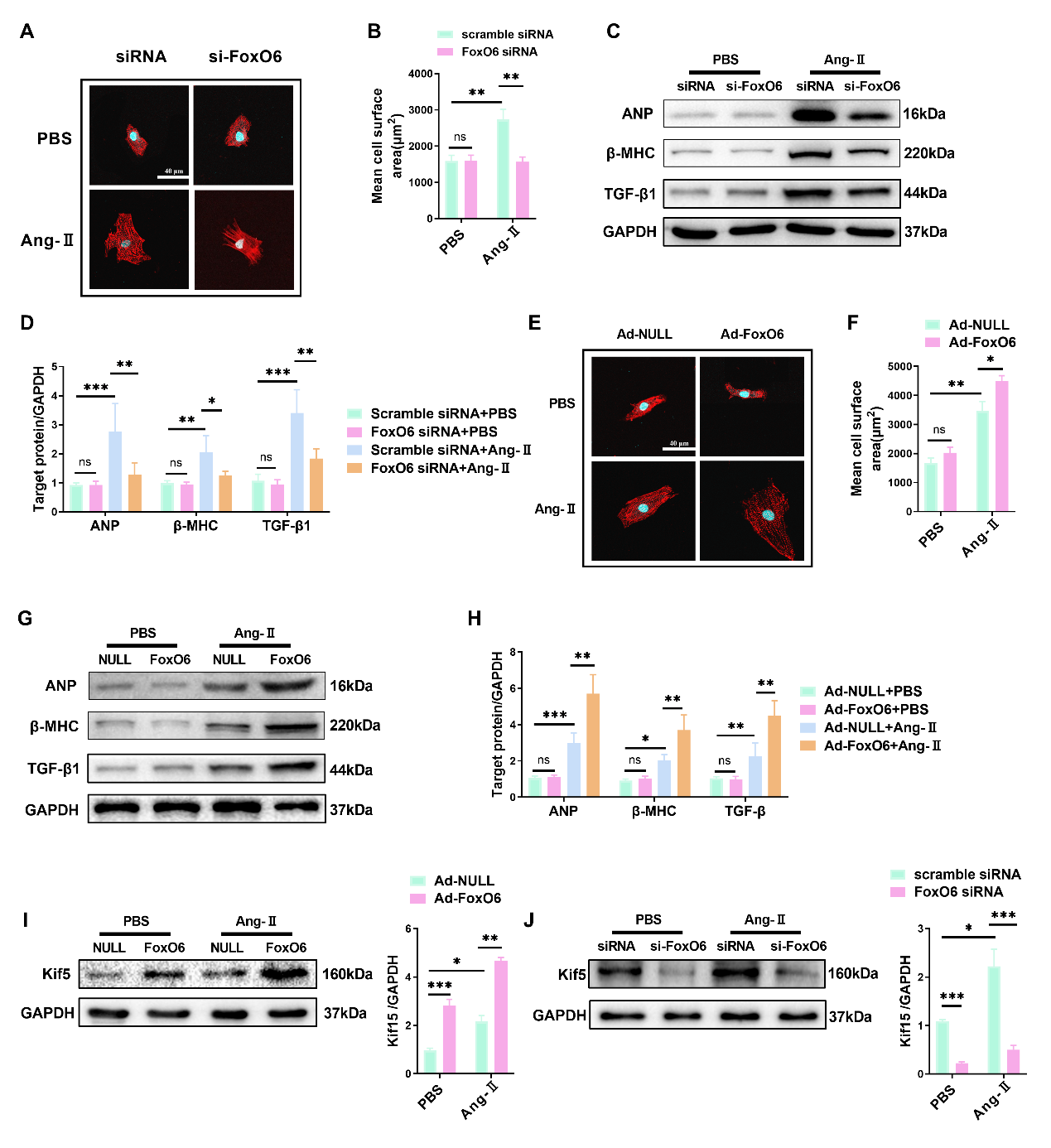


**Figure S6.** (**A**) Immunostaining of NRCMs after Ang-Ⅱ treatment to exhibit the expression of α-actinin (red) and DAPI (blue). (**B**) Mean cell surface area of NRCMs (n ≥ 30 cells/group). (**C**) Typical western blots indicating β-MHC, ANP, and TGF-β1. (**D**) CM protein expression levels of β-MHC, ANP, and TGF-β1 (n = 5). (**E**) Immunostaining of NRCMs after Ang-Ⅱ treatment to exhibit the expression of α-actinin (red) and DAPI (blue). (**F**) Mean cell surface area of NRCMs (n ≥ 30 cells/group). (**G**) Typical western blots indicating β-MHC, ANP, and TGF-β1. (**H**) CM protein expression levels of β-MHC, ANP, and TGF-β1 (n = 5). (**I–J**) CM protein expression levels of Kif15 (n = 5). Data were analyzed by one-way ANOVA. *p < 0.05, **p < 0.01, ***p < 0.001, ****p < 0.0001. Statistics are carried out as mean ± standard deviation (SD).


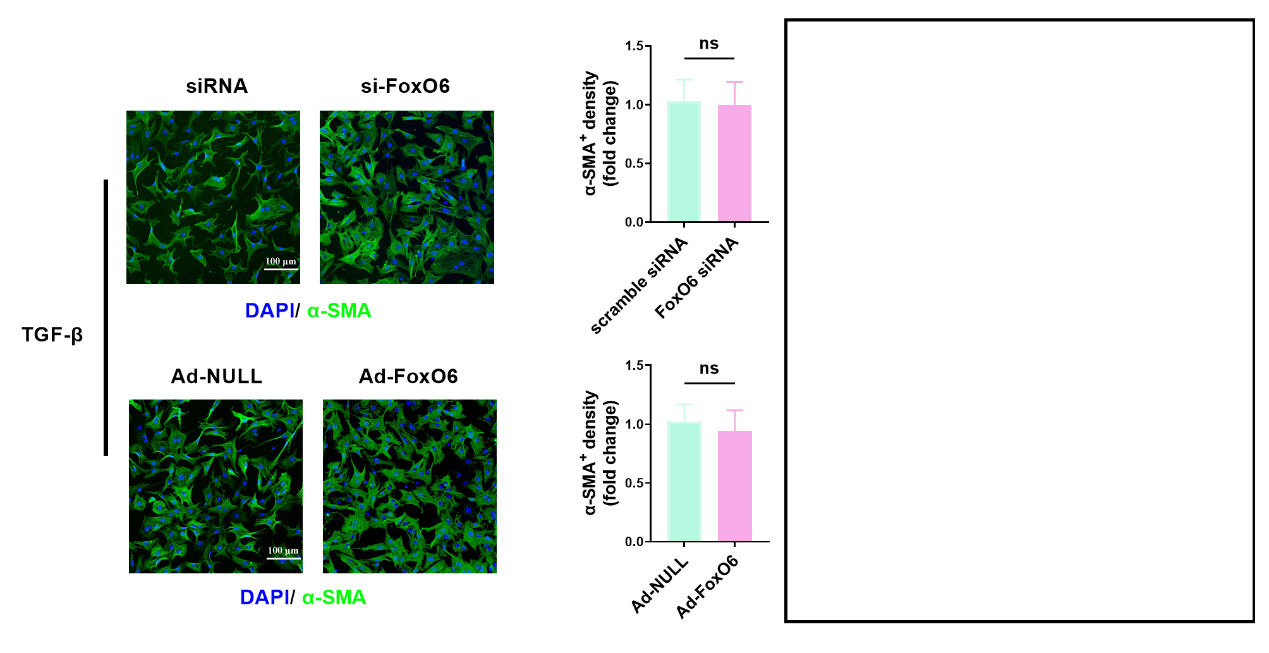


**Figure S7. Left:** Representative immunofluorescence images of fibroblasts exposed to TGF-β1 stained with α-SMA (green) and DAPI (blue) and observed under confocal microscope. **Right:** α-SMA immunofluorescence intensity (n = 5 samples per group). Data were analyzed by *t*-test. ^ns^p > 0.05. Statistics are carried out as mean ± standard deviation (SD).


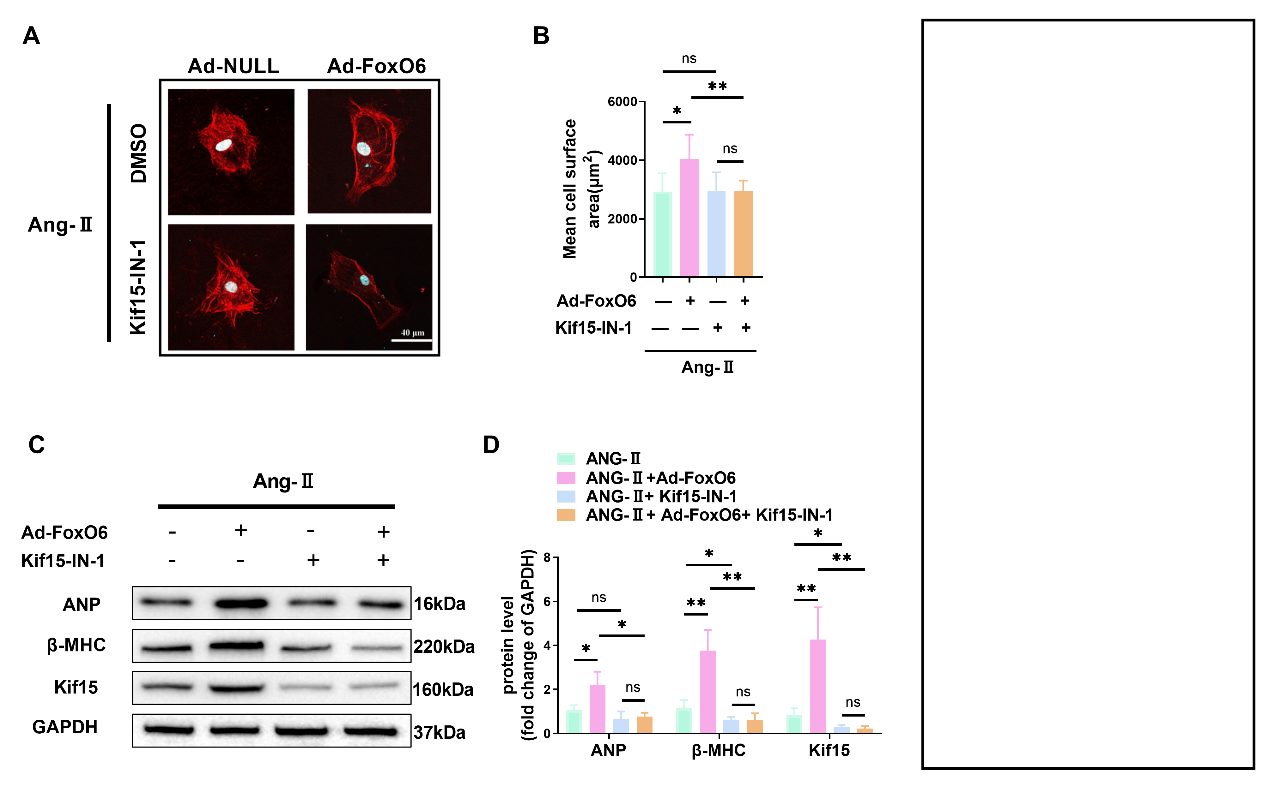


**Figure S8.** (**A**) Immunostaining of NRCMs after Ang-Ⅱ treatment to exhibit the expression of α-actinin (red) and DAPI (blue). (**B**) Mean cell surface area of NRCMs (n ≥ 30 cells/group). (**C**) Typical western blots indicating β-MHC, ANP, and TGF-β1. (**D**) CM protein expression levels of β-MHC, ANP, and TGF-β1 (n = 5). Data were analyzed by one-way ANOVA. *p < 0.05, **p < 0.01. Statistics are carried out as mean ± standard deviation (SD).


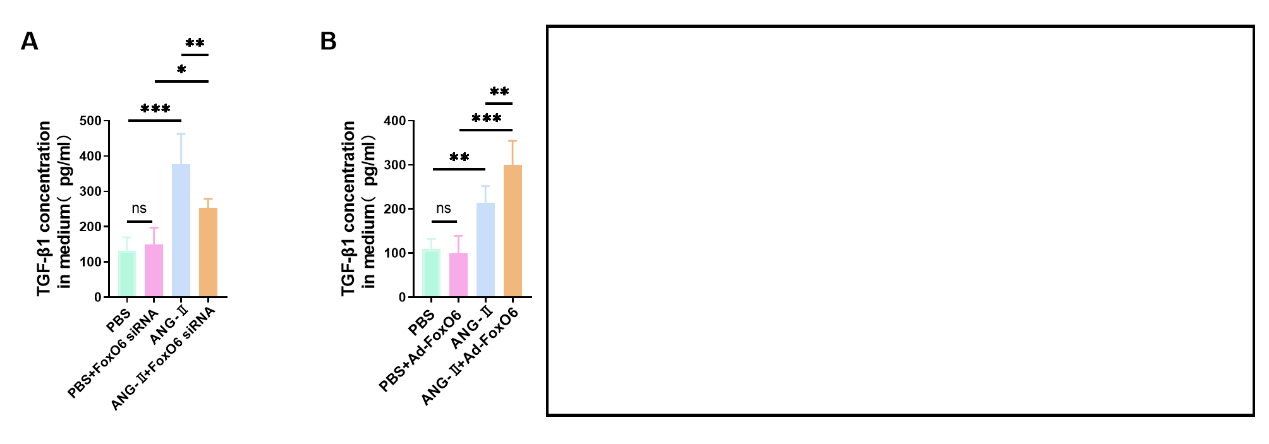


**Figure S9.** (**A–B**) TGF-β1 concentration in media from different groups (n = 5 samples per group). Data were analyzed by one-way ANOVA. ^ns^p > 0.05, *p < 0.05, **p < 0.01, ***p < 0.001, ****p < 0.0001. Statistics are carried out as mean ± standard deviation (SD).


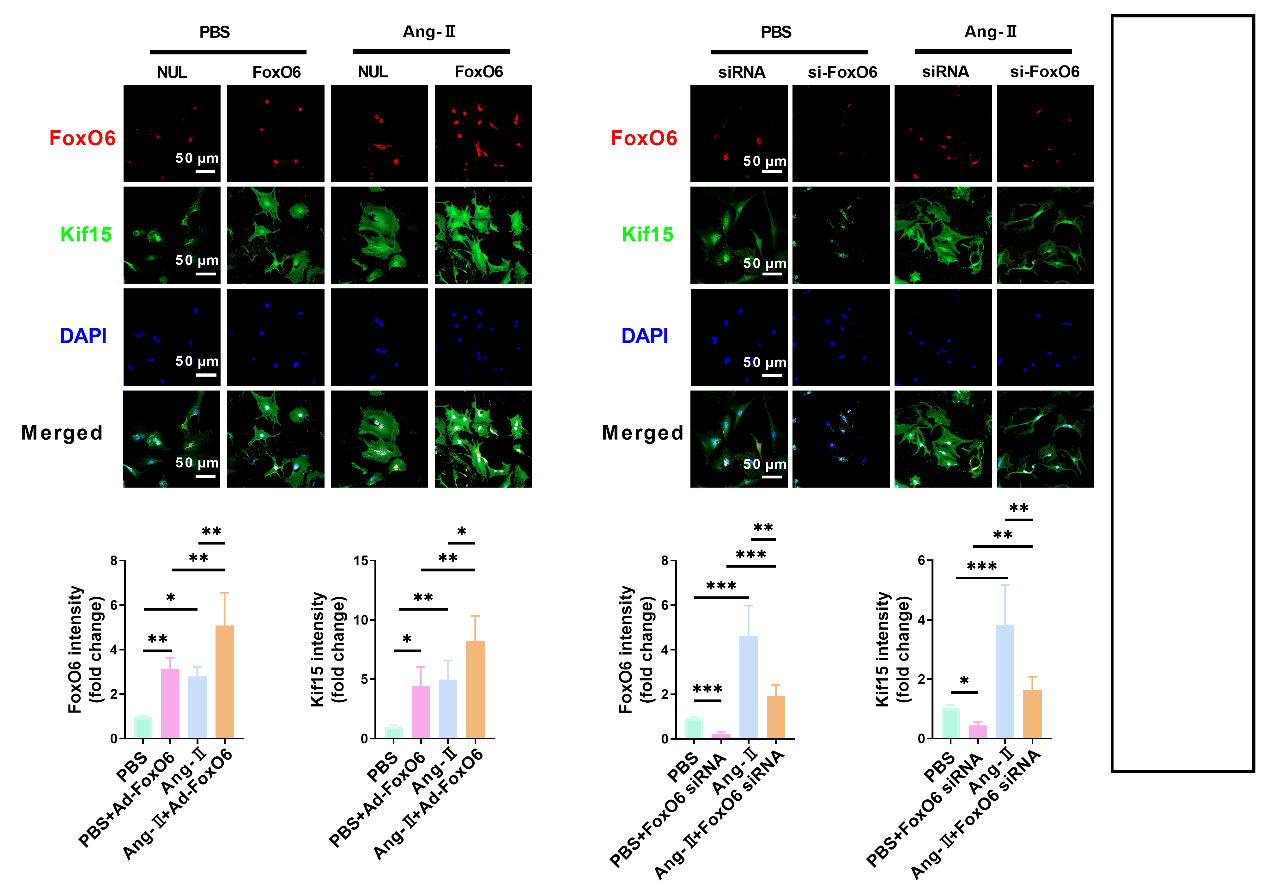


**Figure S10. Above:** Immunostaining of NRCMs to exhibit the expression of FoxO6 (red), Kif15 (green), and DAPI (blue). **Below:** FoxO6 and Kif15 immunofluorescence intensity (n = 5 samples per group). Data were analyzed by one-way ANOVA. ^ns^p > 0.05, *p < 0.05, **p < 0.01, ***p < 0.001, ****p < 0.0001. Statistics are carried out as mean ± standard deviation (SD).


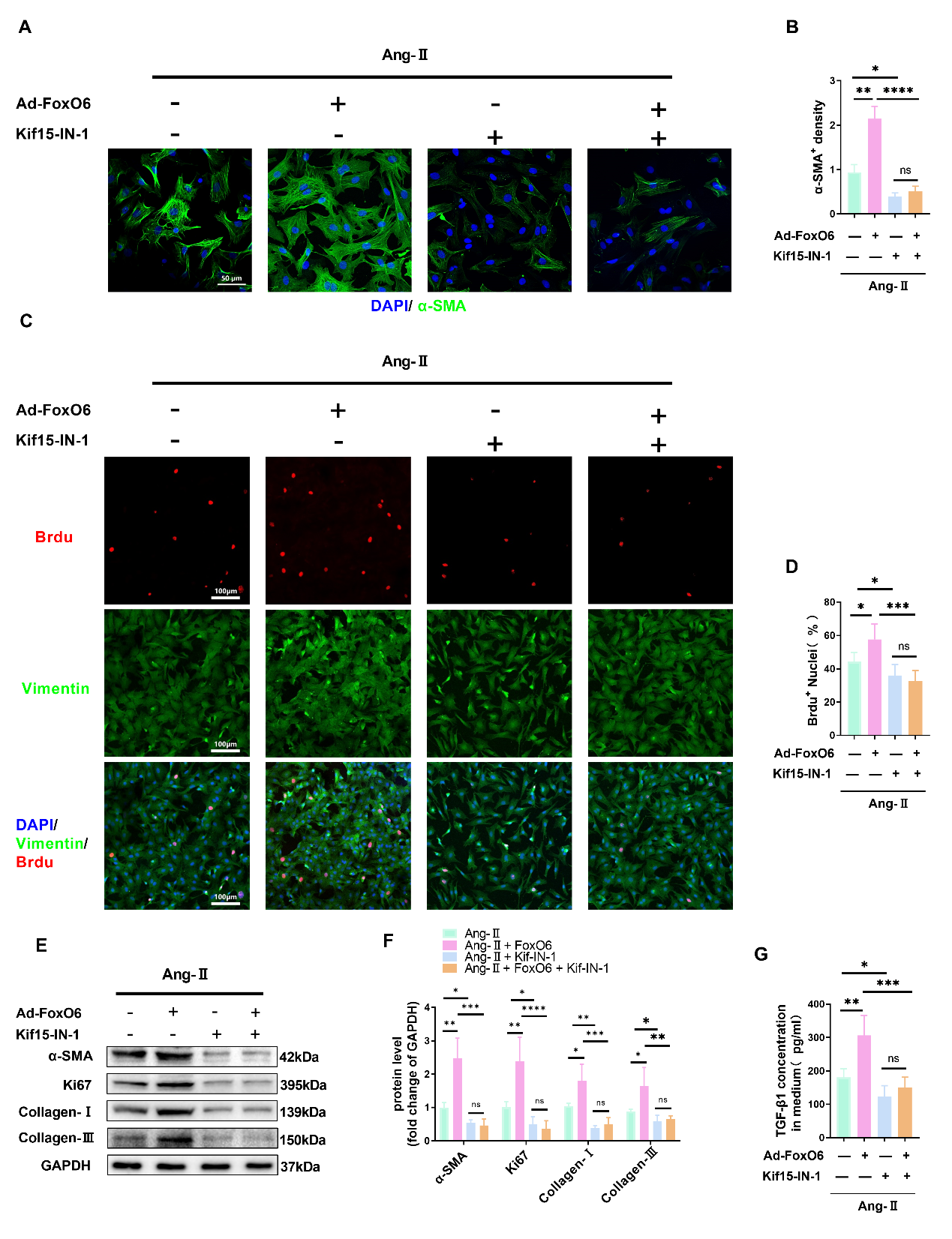


**Figure S11.** (**A**) Immunostaining of fibroblasts to exhibit the expression of α-SMA (green) and DAPI (blue). (**B**) α-SMA immunofluorescence intensity (n = 5 samples per group). (**C**) Immunostaining of fibroblasts to exhibit the expression of Vimentin (green), Brdu (red), and DAPI (blue). (**D**) Number of Brdu-positive nuclei (n = 5). (**E**) Typical western blots indicating α-SMA, Ki67, collagen-Ⅰ, and collagen-III. (**F**) FB protein expression levels of α-SMA, Ki67, collagen-Ⅰ, and collagen-III (n = 6). (**G**) TGF-β1 concentration in media from different groups (n = 6). Data were analyzed by one-way ANOVA. ^ns^p > 0.05, *p < 0.05, **p < 0.01, ***p < 0.001, ****p < 0.0001. Statistics are carried out as mean ± standard deviation (SD).


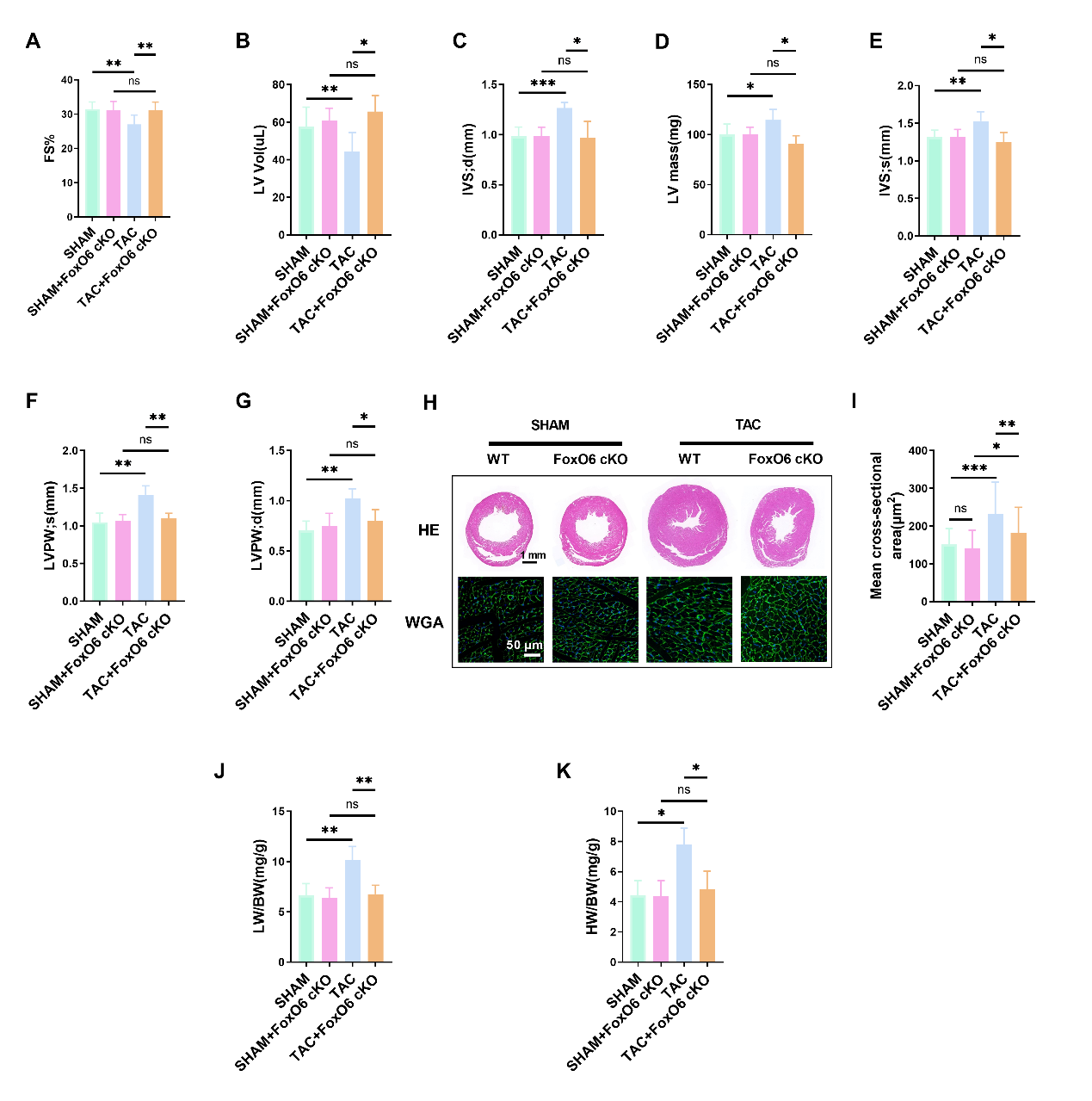


**Figure S12. (A–G**) FS%, LV vol, IVSd, LV mass, IVSs, LVPWs, and LVPWd, respectively, determined via echocardiography. (**H**) Typical images of sections stained with HE and WGA. (**I**) Analysis of CMs cross-sectional area (n ≥ 100 cells/group). (**J**) LW/BW ratio of mice (n = 8). (**I**) HW/BW ratio of mice (n = 8). Data were analyzed by one-way ANOVA. ^ns^p > 0.05, *p < 0.05, **p < 0.01, ***p < 0.001, ****p < 0.0001. Statistics are carried out as mean ± standard deviation (SD).

**Table S1. Primers used in this study.**

| **Gene** | **Forward primer** | **Reverse primer** |
| --- | --- | --- |
| *Gapdh (mouse)* | AGAACATCATCCCTGCATCC | AGTTGCTGTTGAAGTCGC |
| *β-mhc (mouse)* | ACTGTCAACACTAAGAGGGTCA | TTGGATGATTTGATCTTCCAGGG |
| *Anp (mouse)* | TCTTCCTCGTCTTGGCCTTT | CCAGGTGGTCTAGCAGGTTC |
| *Bnp (mouse)* | TGGGAGGTCACTCCTATCCT | GGCCATTTCCTCCGACTTT |
| *Acta-1 (mouse)* | CCAAAGCTAACCGGGAGAAG | GACAGCACCGCCTGGATAG |
| *Rcan1.4 (mouse)* | TTGTGTGGCAAACGATGATGT | CCCAGGAACTCGGTCTTGT |
| *Tgfβ1 (mouse)* | GAGCCCGAAGCGGACTACTA | TGGTTTTCTCATAGATGGCGTTG |
| *α-sma (mouse)* | GCCCAGAGCAAGAGAGG | TGTCAGCAGTGTCGGATG |
| *Collagen-Ⅰ (mouse)* | CTGGCGGTTCAGGTCCAAT | TTCCAGGCAATCCACGAGC |
| *Collagen-Ⅲ (mouse)* | TGAATGGTGGTTTTCAGTTCAG | GATCCCATCAGCTTCAGAGACT |
| *Postn (mouse)* | CACCTGTGAACAATGCGT | CAGATAGCCGTCCGATACA |
| *Foxo6 (mouse)* | CGGGCTAGGTGGGAGTG | TGGAGTTGGGTGGCTTAGG |
| *Kif15 (mouse)* | AACACCACCCCATTTTCA | CCAGGCGTAGATTTTCCA |
| *Gapdh (rat)* | TGACAACTCCCTCAAGATTGTCA | GGCATGGACTGTGGTCATGA |
| *Foxo6 (rat)* | ATGTCAGCCTCCGCCAA | GCCTCGTCGTCCTCCAG |
| *Kif15 (rat)* | TACCACTCCAACCAAGGC | TCTGTGAGCGCAGGAAG |
| *Tgfβ1 (rat)* | CCTACATTTGGAGCCTGGA | CCGGGTTGTGTTGGTTG |
